# Changes in dietary sodium knowledge, attitudes and behaviors among Canadian adults in 2011 and 2024: a repeated cross-sectional study

**DOI:** 10.1016/j.ajcnut.2026.101213

**Published:** 2026-01-27

**Authors:** Rola Al Ghali, Michael Prashad, Wendy Lou, Mary L’Abbe, JoAnne Arcand

**Affiliations:** 1 Department of Nutritional Sciences, The Faculty of Health Sciences, Ontario Tech University, Oshawa, Ontario, L1G 0C5, Canada; 2 Department of Statistics, Dalla Lana School of Public Health, University of Toronto, Toronto, Ontario, M5T 3M7, Canada; 3 Faculty of Medicine, Department of Nutritional Sciences, University of Toronto, Toronto, ON, Canada

**Keywords:** survey, sodium, knowledge, attitudes, and behaviors, repeated cross-sectional study, Canadian adults

## Abstract

**Background:**

Excess sodium intake is a leading modifiable risk for noncommunicable diseases. Despite some national sodium-reduction initiatives, no population-level updates to sodium-related knowledge, attitudes, behaviors (KAB) have been available since 2011.

**Objectives:**

This study aimed to compare sodium-related KAB among Canadian adults in 2011 and 2024 and examine differences by sex and age, hypothesizing limited changes due to insufficient national sodium-reduction initiatives.

**Methods:**

Repeated cross-sectional national surveys were conducted in 2011 (*n* = 2603) and 2024 (*n* = 3267), readministering the same sodium KAB questions from the 2011 survey. Knowledge responses were coded as correct/incorrect, and 5-point Likert-scale items were recoded or dichotomized. Data were weighted to the Canadian census. Rao–Scott adjusted χ^2^, *t*-tests and regression models assessed changes over time, stratifying by sex and age.

**Results:**

The proportion of adults actively limiting sodium intake declined from 57.4% in 2011 to 37.3% in 2024 (*P* < 0.001). Concurrently, engagement in nearly all sodium-reduction behaviors decreased in 2024, including avoiding processed foods (69.3%–52.3%), not adding salt at the table (69.2%–58.1%), avoiding salt during cooking (62.3%–43.4%) (all *P* < 0.001), and reading Nutrition Facts labels (54.2%–49.6%, *P* = 0.005). Paradoxically, overall population sodium concern remained high and unchanged (66.3%–65.9%, *P* = 0.812). Food label interpretation and awareness of recommended sodium intake improved (*P* < 0.001), but knowledge of health-related conditions linked to sodium such as blood pressure, heart disease, and stroke decreased (all *P* < 0.001). Reported barriers to sodium reduction included cost, taste, time constraints, and lack of social support.

**Conclusions:**

Many indices of sodium-related KAB deteriorated from 2011 to 2024 among Canadian adults, despite their continued concern about sodium intake. These findings highlight a widening knowledge-to-action gap and reinforce the need for comprehensive public health efforts to support population-wide dietary sodium reduction.

## Introduction

Noncommunicable diseases account for >70% of global deaths worldwide, with high-sodium intake being one of the leading dietary risk factors [1]. The consumption of dietary sodium above recommended levels elevates blood pressure and increases the risk of hypertension, cardiovascular disease, stroke, and kidney disease [[2], [3], [4], [5], [6]]. In Canada, approximately one-third of hypertension is attributable to high dietary sodium intake, affecting about 2 million Canadians and contributing to >12,000 deaths and >150,000 disability-adjusted life years annually [7]. The WHO recommends limiting daily sodium intake to <2000 mg/d, a threshold associated with significant reductions in blood pressure and improved cardiovascular outcomes in people with and without hypertension [2,6,8]. Despite strong evidence, sodium intake remains well above recommended levels in most countries, including Canada [8]. In many countries, like Canada, the United States, and those in western Europe, the majority of dietary sodium is derived from processed and restaurant foods rather than discretionary salt [[8], [9], [10]]. Modeling studies suggest that reducing sodium intake to recommended levels in Canada could prevent or delay 5296 (9.1%) of cardiovascular deaths annually, reduce hypertension prevalence by 30%, and save $1.38 billion in annual healthcare costs [11,12].

In response to concerns about excess dietary sodium, Canada has implemented several sodium-reduction initiatives aligned with WHO targets [8,13], including voluntary sodium-reduction targets for processed foods, updated dietary guidelines, and forthcoming front-of-package warning labels (FOPL) [[14], [15], [16]]. Several provinces have also introduced sodium-related procurement policies, and sodium has been featured within the Healthy Eating Strategy and the 2019 Canada’s Food Guide [[17], [18], [19]]. However, national sodium intake has remained largely unchanged [10,20], and there is consensus that observed declines in dietary sodium between 2004 and 2015 were likely driven by underreporting rather than actual behavior change [10,21]. Contributing factors include limited industry compliance with voluntary reformulation targets, the absence of sustained sodium-focused public health education campaigns, and competing nutrition messages [10,22,23].

Routine monitoring of sodium-related knowledge, attitudes, and behaviors (KAB) is recommended by the WHO as part of national sodium-reduction programs [24,25]. KAB data help identify knowledge gaps, actions and related barriers, misconceptions and population subgroups requiring tailored intervention. However, the most recent national sodium KAB study in Canada was conducted in 2011 [26], leaving a 13-y gap in surveillance despite some changes in the food environment, digital information landscapes, and national nutritional policies. Therefore, this study compared sodium KAB among Canadian adults in 2011 and 2024. Given the limited impact of existing national sodium-reduction policies and the absence of dedicated sodium-focused awareness campaigns, we hypothesized that only modest improvements in sodium KAB would be observed, and that persistent or widening gaps would reflect challenges in translating awareness into behavior.

## Methods

### Study design

This study used repeated cross-sectional data from 2 national surveys administered to Canadian adults in 2011 and 2024, using a common set of sodium KAB questions to assess changes over time. The study was approved by the Ontario Tech University Research Ethics Board (REB #17207 and 17546). All participants provided written informed consents, and no identifying data were collected. Participants were incentivized through Leger’s LEO program [1000 points = Canadian Dollar (CAD) $1], receiving CAD $2.50 for completing the survey, redeemable for rewards.

### Participants and sample size

**FIGURE 1:**
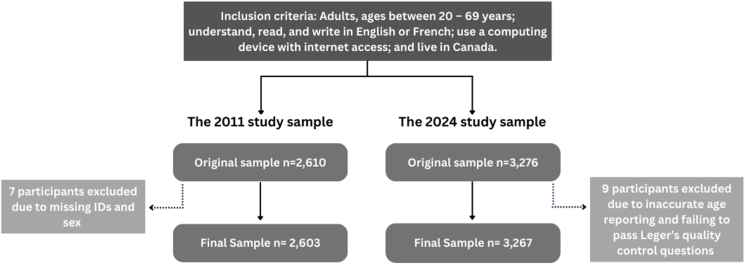
Flow diagram illustrating participant inclusion and exclusion criteria leading to the final analytical samples for the 2011 and 2024 surveys. ID, identity document.

The inclusion criteria were identical in 2011 and 2024. Eligible participants were adults aged 20 to 69 y who could understand, read, and write in English or French, used a computing device with internet access, and resided in Canada. In 2011, the online survey was administered to the National Network for Advanced Foods and Materials survey panel (*n* = 2610) [26]. After applying predefined quality-control checks, 7 participants were excluded for missing sex and identification data (*n* = 7), resulting in an analytical sample of 2603. The 2024 survey was administered online by Leger Marketing, drawing from Leger’s proprietary internet panel of >400,000 Canadian adults profiled on sociodemographic characteristics. Responses were required to all questions except for those with programmed skip logic. Of 3276 responses, 9 responses were excluded for reporting a biologically implausible age (*n =* 3) or for failure to correctly answer embedded data-quality questions (*n =* 6), yielding a final analytical sample of 3267 participants (Figure 1). A sample of 2603 participants provided >80% power at a 1% significance level to detect a minimum 3% increase in knowledge of recommended sodium intake from 2011 to 2024 [26]. This sample size also provided 95% power to detect a 5% increase. The 2024 sample of *n* = 3267 exceeded this threshold.

### Measures

#### Identification of common KAB variables

A total of 24 questions assessing sodium KAB, many evaluating multiple statements, were included in both the 2011 and 2024 surveys. Questions captured knowledge of sodium recommendations and health effects, concerns, behavioral strategies to reduce sodium, perceived reasons and perceived barriers for limiting/not limiting sodium. The 2011 survey underwent content and face validation by 7 experts in sodium, public health, and nutrition policy [26]. To guide retention decisions, experts evaluated each question in the 2011 survey using a defined scoring system, with each item being rated as “Include” = 1, “Consider including” = 2, or “Exclude” = 3. Questions with a mean score of ≥1.5 (≥75%) were retained; those scoring between 1.2 and 1.49 (60%–74.5%) were considered for inclusion; and those with scores <1.2 (<60%) were excluded. Identical question wording and response formats were preserved to maximize comparability over time [27] (Supplemental Appendix 1).

#### Outcome variables

Knowledge was assessed using multiple-choice questions that included an “I do not know” option, which was coded as an incorrect response (0). Behavioral and attitudinal items used 5-point Likert scales (1 = Strongly disagree/Never do this; 5 = Strongly agree/Always do this), with most having a “Not applicable” or “I do not know” option. Items assessing reasons for limiting or not limiting sodium intake incorporated skip logic. Certain variables were dichotomized: sodium-reduction behaviors were coded as *doing the behavior* (responses 4–5: strongly agree/agree) versus *not doing the behavior* (responses 1–3: strongly disagree/disagree /neutral); concern about sodium was dichotomized as *expressing concern* (extremely, very, or somewhat concerned) compared with *not expressing concern* (not very or not at all concerned); and engagement in sodium reduction was dichotomized as *taking action* (“I am currently trying”) versus *not taking action* (“I am not limiting my intake,” “I am interested but have not started,” or “I have tried, but not anymore.”) The 2024 survey included an “intersex” response option (*n =* 6). Because the sample size was insufficient for inferential testing, and because we did not prespecify hypotheses for this group, these cases were redistributed proportionally across female and male categories for the statistical analyses. Sensitivity tests excluding these participants yielded identical results.

### Statistical analysis

Knowledge items were treated as binary outcomes (correct and incorrect). Likert-scale questions were analyzed either as categorical variables [recoded into 3 categories: “agree” (strongly agree/agree), “disagree” (strongly disagree/disagree), and “neutral” (retained as a separate category)] or as continuous variables. These were summarized, respectively, using weighted frequencies and percentages, or weighted mean scores, to capture overall response trends. Primary outcomes were differences in sodium KAB in 2011 and 2024. Explanatory variables included survey year, sex (females and males), and age group (20–29, 30–39, 40–49, 50–59, 60–69 y). All estimates were weighted to reflect the 2006 and 2021 Canadian census populations, respectively, by age, sex and province/territory. Responses excluded from analysis were classified a priori into 4 categories, based on survey design and response meaning: *1*) *not applicable* responses; *2*) “I do not know” responses; *3*) responses omitted due to *planned skip logic*; and *4*) *item nonresponse*. Primary analyses were conducted using complete-case data among eligible respondents. As a sensitivity analysis, “I do not know” responses (3.1%–5.0% in 2011 and 6.7%–11.4% in 2024) and item nonresponse (0.2%–5.3% only in 2011) were treated as missing data (Supplemental Table 1) and addressed using multiple imputation. Results from imputed and complete-case analyses were compared with assess robustness.

Descriptive analyses reported categorical data as weighted frequencies and percentages with 95% confidence intervals (CIs). To assess change relative to baseline in 2011, relative percentage change (RPC) was calculated as: RPC = (2024–2011)/2011 [28]. Likert-scale variables were presented as weighted mean differences (2024–2011) with 95% CIs. Differences from 2011 to 2024, overall and within sex and age groups, were tested using Rao–Scott adjusted χ^2^ tests for categorical and dichotomized variables, and survey-weighted *t*-tests for continuous variables. Survey-weighted logistic regression was used to estimate odds ratios (OR) for dichotomized outcomes, and survey-weighted linear regression for continuous (mean-scaled) Likert variables. All models controlled for age group and sex, to account for sociodemographic differences, with survey year as the primary exposure. These covariates were selected a priori based on prior literature [26]; no data-driven variable selection procedures were applied. For all regression analyses, females and the youngest age group (20–29 y) served as reference categories. Interaction terms were included to assess sex-by-year and age-by-year effects. Given the descriptive nature of this study and the large number of outcomes examined, a conservative significance threshold (*P* < 0.01) was used for primary comparisons. Bonferroni adjustments were applied to post hoc comparisons, with a target familywise error rate of 0.01. Additionally, the findings were interpreted with emphasis on effect size and direction [29]. All analyses were conducted in RStudio (version 2025.05.0).

## Results

After exclusions for missing or invalid data, the final analytical samples included 2603 participants in 2011 and 3267 in 2024 (Figure 1). Weighted demographic characteristics of participants from both survey years are presented in Table 1. The samples were similar by sex (51% female in both years) and showed slightly greater representation of adults aged 60 to 69 y in 2024 (13.6% in 2011; 21.3% in 2024). Geographic distribution was consistent across survey years with Ontario (∼38%) and Quebec (∼24%), the most populated provinces, being the most represented. Educational attainment was similar across survey years, with the 2024 sample having slightly more university-educated (37.9% in 2011; 41.5% in 2024) and fewer college-educated participants (34.9% in 2011; 30.4% in 2024). Approximately one-fifth of participants in each cohort reported having been advised by a healthcare provider to reduce sodium intake.

**TABLE 1:** Weighted demographic characteristics of the 2011 and 2024 study populations

	2011 (%) (*n* = 2603)	2024 (%) (*n* = 3267)
Sex		
Females	51.0	51.4
Males	49.0	48.6
Age (y)		
20–29	19.4	17.6
30–39	20.4	22.1
40–49	25.2	20.1
50–59	21.4	19.0
60–69	13.6	21.3
Province/territory		
Alberta	10.5	11.3
British Columbia	13.2	13.8
Manitoba	3.5	3.5
New Brunswick	2.2	2.0
Newfoundland	1.7	1.4
Nova Scotia	2.9	2.8
Ontario	38.4	38.0
Prince Edward Island	0.4	0.5
Quebec	24.4	23.1
Saskatchewan	2.9	3.3
Territories	0.0	0.5
Highest education level		
<High school	1.3	1.4
High school	16.9	19.3
Trade school	9.1	7.4
College	34.9	30.4
University	37.9	41.5
Parent/caregiver	38.0	28.4
Diagnosed with hypertension	21.1	23.7
Advised by medical professional to limit sodium intake	20.1	21.2

All estimates were weighted to reflect the 2006 and 2021 Canadian census populations, respectively, considering age, sex, and province/territory.

### Changes in action to limit dietary sodium

From 2011 to 2024, the proportion of participants reporting that they were taking action to reduce their sodium intake decreased from 57.4% to 37.3% (*P* < 0.001; 35.1% relative decrease) (Table 2). No significant differences in action-taking were observed by sex over time. However, engagement in sodium-reduction actions declined significantly across all age groups (*P* = 0.002) (Supplemental Table 2).

**TABLE 2:** Changes in action and reported reasons for limiting or not limiting dietary sodium, 2011 and 2024

	2011 (*n* = 2603)		2024 (*n* = 3267)		RPC/MD	*P* value
	%/MS	95% CI	%/MS	95% CI		
Taking action to reduce sodium (%)	57.4	(54.8, 60.0)	37.3	(35.6, 39.0)	–35.1	<0.001
Reasons for limiting sodium						
Managing a current health condition						
Disagree (%)	35.7	(32.5, 38.9)	20.1	(18.3, 22.0)	–43.7	—
Neutral (%)	18.4	(16.0, 21.1)	23.6	(21.7, 25.6)	28.3	<0.001
Agree (%)	45.9	(42.8, 49.1)	56.4	(54.1, 58.6)	22.9	—
MS	3.2	(3.1, 3.3)	3.5	(3.4, 3.5)	0.3 (0.2, 0.4)	<0.001
Disagree (%)	49.5	(46.3, 52.6)	36.2	(33.9, 38.5)	–26.9	—
Neutral (%)	19.3	(17.1, 21.8)	24	(22.0, 26.1)	24.4	<0.001
Agree (%)	31.2	(28.5, 34.1)	39.8	(37.5, 42.1)	27.6	—
MS	2.7	(2.6, 2.8)	3.0	(2.9, 3.0)	0.3 (0.1, 0.4)	<0.001
Reasons for not limiting sodium						
Believing sodium is not bad to health						
Disagree (%)	48.7	(43.1, 54.3)	38.9	(35.5, 42.4)	–20.1	—
Neutral (%)	30.3	(25.4, 35.6)	35.7	(32.4, 39.2)	17.8	0.014
Agree (%)	21.1	(16.9, 25.9)	25.4	(22.4, 28.6)	20.4	—
MS	2.6	(2.5, 2.7)	2.8	(2.7, 2.9)	0.2 (0.0, 0.3)	0.010
Conflicting information about sodium and health						
Disagree (%)	50.2	(44.3, 56.0)	38.5	(34.9, 42.3)	–23.3	—
Neutral (%)	32	(26.9, 37.4)	36.6	(33.0, 40.3)	14.4	0.003
Agree (%)	17.9	(13.9, 22.8)	24.9	(21.8, 28.3)	39.1	—
MS	2.5	(2.3, 2.6)	2.8	(2.7, 2.9)	0.3 (0.1, 0.5)	0.001
Having normal or low blood pressure						
Disagree (%)	10.2	(7.3, 14.0)	13.0	(10.8, 15.6)	27.5	—
Neutral (%)	16.4	(12.8, 20.7)	22.4	(19.6, 25.5)	36.6	0.017
Agree (%)	73.5	(68.4, 78.0)	64.6	(61.2, 67.9)	–12.1	—
MS	4.0	(3.9, 4.2)	3.8	(3.7, 3.9)	−0.2(−0.4, −0.1)	0.002
Taking blood pressure medication						
Disagree (%)	77.2	(70.6, 82.8)	60.4	(55.9, 64.7)	–21.8	—
Neutral (%)	15.8	(11.2, 22.0)	22.0	(18.5, 25.9)	39.2	0.001
Agree (%)	6.9	(4.2, 11.3)	17.6	(14.4, 21.3)	155.1	—
MS	1.6	(1.5, 1.8)	2.2	(2.1, 2.3)	0.6 (0.4, 0.7)	<0.001
Not recommended by healthcare provider						
Disagree (%)	27.8	(22.4, 33.8)	13.8	(11.4, 16.8)	–50.4	—
Neutral (%)	17.9	(12.9, 24.4)	21.7	(18.7, 25.0)	21.2	<0.001
Agree (%)	54.3	(47.7, 60.8)	64.5	(60.7, 68.1)	18.8	—
MS	3.5	(3.3, 3.6)	3.8	(3.7, 3.9)	0.3 (0.1, 0.5)	0.001

Abbreviations: CI, confidence interval; MD, mean difference; MS, mean score; RPC, relative percentage change.

Several motivations for reducing sodium intake shifted over time (Table 2 and Supplemental Table 2). Participants in 2024 were more likely to report reducing dietary sodium to manage a current health condition (45.9%–56.4% agreeing; mean score = 3.2–3.5; mean difference = 0.3, 95% CI: 0.2, 0.4; *P* < 0.001) or because of a healthcare provider recommendation (31.2%–39.8% agreeing; mean score = 2.7–3.0; mean difference = 0.3, 95% CI: 0.1, 0.4; *P* < 0.001). Although the absolute increases were modest, both health-related motivations increased significantly over time, indicating a shift toward more clinically driven reasons for sodium reduction. In contrast, significantly more participants in 2024 reported not limiting sodium intake because it had not been recommended by a healthcare provider (54.3%–64.5% agreeing; mean score = 3.5–3.8; mean difference = 0.3; 95% CI: 0.1, 0.5; *P* < 0.001), citing conflicting information about sodium and health (17.9%–24.9% agreeing; mean score = 2.5–2.8; mean difference = 0.3, 95% CI: 0.1, 0.5; *P* = 0.001), or because they were taking blood pressure medication (6.9%–17.6% agreeing; mean score = 1.6–2.2; mean difference = 0.6, 95% CI: 0.4, 0.7; *P* = 0.001). The increase in reliance on medication as a perceived substitute for dietary sodium reduction is a clinically important observation and was found to be more common among males (mean score = 1.6–2.4; mean difference = 0.8; 95% CI: 0.5, 1.1; *P* < 0.001), despite the relatively small absolute percentages. The belief that sodium is not bad to health, as a reason for not limiting intake, remained unchanged in 2011 and 2024 (21.1%–25.4% agreeing; mean score = 2.6–2.8; mean difference = 0.2, 95% CI: 0.0, 0.3; *P* = 0.010). However, it is worth noting that one in 4 Canadians holds this belief.

### Changes in attitudes and concern about dietary sodium

Overall concern about sodium intake was similar in 2011 and 2024 (66.3% in 2011 to 65.9% in 2024, RPC = –0.5, *P* = 0.812). There was a general decline in concern about sodium from 2011 to 2024 with increasing age (*P* < 0.001), especially among those aged 50 to 59 y (OR = 0.66; 95% CI: 0.56, 0.77; *P* = 0.005). In contrast, more participants in 2024 believed that dietary sodium reduction improves health (40.2%–48.9%, RPC = 21.6, *P* < 0.001). Fewer participants perceived their sodium intake as the “right amount” (57.0%–47.5%, RPC = –16.7, *P* < 0.001) or lower than mean (62.5%–51.8%, RPC = –17.1, *P* < 0.001), whereas more perceived their personal intake as “too high” (33.4%–47.5%, RPC = 42.2, *P* < 0.001) or “about the same” as the average Canadian (25.1%–36.7%, RPC = 46.2, *P* < 0.001) (Table 3 and Supplemental Table 3).

**TABLE 3:** Changes in attitudes and concern about sodium intake, 2011 and 2024

	2011 (*n* = 2603)		2024 (*n* = 3267)		RPC/MD	*P* value
	%/MS	95% CI	%/MS	95% CI		
Concerned about personal sodium intake						
	66.3	(63.8, 68.7)	65.9	(64.2, 67.5)	–0.5	0.812
Concerned about child’s sodium intake						
	37.1	(33.3, 41.0)	56.6	(53.3, 59.8)	52.6	<0.001
Perception about personal sodium intake						
Too much (%)	33.4	(31.0, 35.8)	47.5	(45.7, 49.3)	42.2	—
The right amount (%)	57.0	(54.4, 59.5)	47.5	(45.7, 49.3)	–16.7	<0.001
Too little (%)	9.7	(8.1, 11.5)	5.0	(4.3, 5.9)	–48.5	—
MS	2.8	(2.7, 2.8)	2.5	(2.5, 2.6)	–0.2 (–0.3, –0.2)	<0.001
Comparing personal intake with average Canadian						
Lower (%)	62.5	(59.9, 65.0)	51.8	(50.0, 53.6)	–17.1	—
About the same amount (%)	25.1	(22.9, 27.4)	36.7	(34.9, 38.4)	46.2	<0.001
Higher (%)	12.4	(10.7, 14.5)	11.6	(10.5, 12.8)	–6.5	—
MS	2.3	(2.3, 2.4)	2.5	(2.5, 2.5)	0.2 (0.1, 0.2)	<0.001
Belief that sodium reduction improves health						
Disagree (%)	28.5	(26.3, 30.8)	17.8	(16.6, 19.2)	–37.5	—
Neutral (%)	31.3	(29.0, 33.7)	33.3	(31.7, 34.9)	6.0	<0.001
Agree (%)	40.2	(37.7, 42.8)	48.9	(47.1, 50.6)	21.6	—
MS	3.2	(3.1, 3.3)	3.4	(3.4, 3.5)	0.2 (0.2, 0.3)	<0.001

Abbreviations: CI, confidence interval; MD, mean difference; MS, mean score; RPC, relative percentage change.

### Changes in sodium-related knowledge and misconceptions

Knowledge of the recommended daily maximum sodium intake improved from 12.4% in 2011 to 21.1% in 2024 (*P* < 0.001), representing a relative improvement of 70%. Correct interpretation of sodium content on Nutrition Facts tables also increased (44.4%–55.6%, RPC = 25.2, *P* < 0.001). In contrast, fewer participants in 2024 recognized the overall health impacts of excess sodium (69.1%–63.4%, RPC = –8.2, *P* < 0.001), or correctly identified associated health conditions such as hypertension (93.9%–82.0%, RPC = –12.7, *P* < 0.001), heart disease (86.2%–73.7%, RPC = –14.5, *P* < 0.001), and stroke (78.4%–59.5%, RPC = –24.2, *P* < 0.001). These represent large absolute reductions (12–24 percentage points), indicating meaningful attrition in knowledge despite improvements in label interpretation (Table 4).

**TABLE 4:** Changes in sodium-related knowledge and misconceptions, 2011 and 2024

	2011 (*n* = 2603)		2024 (*n* = 3267)		RPC	*P* value
	%	95% CI	%	95% CI		
Knowing that sodium affects overall health						
	69.1	(66.6, 71.5)	63.4	(61.7, 65.1)	–8.2	<0.001
Associating sodium with:						
Hypertension	93.9	(92.5, 95.1)	82.0	(80.6, 83.3)	–12.7	<0.001
Heart disease	86.2	(84.2, 88.0)	73.7	(72.1, 75.2)	–14.5	<0.001
Stroke	78.4	(76.0, 80.6)	59.5	(57.8, 61.2)	–24.2	<0.001
Osteoporosis	13.8	(12.1, 15.5)	20.5	(19.2, 22.0)	48.6	<0.001
Arthritis	37.1	(34.6, 39.7)	32.6	(31.0, 34.2)	–12.1	<0.001
Diabetes	38.7	(36.1, 41.3)	38.4	(36.7, 40.1)	–0.8	0.854
Depression	42.0	(39.4, 44.7)	39.8	(38.1, 41.5)	–5.2	0.163
Knowing the maximum recommended sodium intake level						
	12.4	(10.8, 14.2)	21.1	(19.7, 22.5)	70.1	<0.001
Correctly interpreting sodium content from nutrition facts table						
	44.4	(41.8, 47.0)	55.6	(53.9, 57.4)	25.2	<0.001
Believing kosher, sea and gourmet salts to be lower in sodium						
Disagree	54.2	(51.6, 56.7)	43.5	(41.7, 45.2)	–19.7	<0.001
Neutral	24.4	(22.3, 26.7)	32.5	(30.9, 34.2)	33.2	
Agree	21.4	(19.4, 23.6)	24.0	(22.6, 25.6)	12.1	
Believing sodium intake is low by not adding salt to food						
Disagree	38.2	(35.8, 40.7)	47.3	(45.6, 49.0)	23.8	<0.001
Neutral	21.2	(19.3, 23.3)	31.2	(29.7, 32.9)	47.2	
Agree	40.5	(38.0, 43.1)	21.5	(20.1, 22.9)	–46.9	
Believing concern about sodium is unnecessary with low or normal blood pressure						
Disagree	75.9	(73.6, 78.0)	59.7	(58.0, 61.4)	–21.3	<0.001
Neutral	15.6	(13.7, 17.6)	25.7	(24.2, 27.3)	64.7	
Agree	8.6	(7.3, 10.1)	14.5	(13.4, 15.8)	68.6	

Abbreviations: CI, confidence interval; RPC, relative percentage change.

Misconceptions also persisted. Fewer participants understood that excess dietary sodium is a concern even for individuals with low or normal blood pressure (75.9%–59.7%, RPC = –21.3, *P* < 0.001) and fewer participants correctly believed that kosher, sea, and gourmet salts have similar sodium content to table salt (54.2%–43.5%, RPC = –19.7, *P* < 0.001). On a positive note, more participants in 2024 recognized that avoiding added salt alone does not mean following a low-sodium diet (38.2%–47.3%, RPC = 23.8, *P* < 0.001) (Table 4). From 2011 to 2024, younger participants, aged 20 to 29 and 30 to 39 y, were less likely to believe that kosher, sea, or gourmet salts have similar sodium content compared with table salt (OR = 0.41; 95% CI: 0.25, 0.68; *P* < 0.001 and OR = 0.51; 95% CI: 0.36, 0.73; *P* < 0.001; respectively). Likewise, across all age groups, participants were less likely to identify that sodium remains a concern for individuals with low or normal blood pressure. The greatest decline was observed among those aged 20 to 29 y (OR = 0.28; 95% CI: 0.15, 0.50; *P* < 0.001) and the smallest decline among those aged 50 to 59 and 60 to 69 y (OR = 0.66; 95% CI: 0.47, 0.92; *P* = 0.002 and OR = 0.59; 95% CI: 0.40, 0.86; *P* = 0.008). No significant differences in sex were detected over time (Supplemental Table 4).

### Changes in behaviors to reduce dietary sodium

From 2011 to 2024, there was a general decline in engagement in sodium-reduction behaviors. Fewer participants in 2024 reported avoiding purchasing or consuming a high-sodium food (56.0%–41.4%; RPC = –26.2, *P* < 0.001); avoiding salt during cooking (62.3%–43.4%, RPC = –30.3, *P* < 0.001) or at the table (69.2%–58.1%, RPC = –16.0, *P* < 0.001); eating less packaged and ready-to-eat foods (69.3%–52.3%, RPC = –24.5, *P* < 0.001); limiting restaurant meals (47.2%–36.7%, RPC = –22.2, *P* < 0.001); using herbs/spices instead of salt (75.9%–60.5%, RPC = –22.2, *P* < 0.001); choosing fresh produce more often (80.6–67.9%, RPC = –15.8, *P* < 0.001); tasting food before adding salt (79.3%–70.5%, RPC = –11.0, *P* < 0.001); and reading Nutrition Facts tables to determine sodium content (54.2%–49.6%, RPC = –8.4, *P* = 0.005). Engagement in some sodium-reduction behaviors remained unchanged, including purchasing low/reduced-sodium products, draining/rinsing canned foods, and checking sodium amounts or % daily value (DV) on labels. In contrast, few sodium-reduction behaviors improved, specifically looking or intending to look for a healthy choice symbol or logo (29.5% in 2011 to 46.2% in 2024, RPC = 56.4, *P* < 0.001); looking or intending to look for a “low sodium” message or claim (38.8%–49.3%, RPC = 27.1, *P* < 0.001); and requesting “no added salt” at restaurants (5.1%–13.7%, RPC = 171.1, *P* < 0.001) (Table 5). Despite large relative percentage increases, absolute engagement in these behaviors remained modest.

**TABLE 5:** Changes in behaviors to reduce dietary sodium from 2011 to 2024

	2011 (*n* = 2603)		2024 (*n* = 3267)		RPC	*P* value
	2011%	95% CI	2024%	95% CI		
Avoided purchasing or consuming a food due to high-sodium content	56.0	(53.4, 58.7)	41.4	(39.6, 43.2)	–26.2	<0.001
Cooking and use of discretionary salt						
Avoiding salt during cooking	62.3	(59.8, 64.8)	43.4	(41.7, 45.2)	–30.3	<0.001
Using spices, herbs and/or seasonings	75.9	(73.6, 78.1)	60.5	(58.8, 62.2)	–20.3	<0.001
Avoiding salt at the table	69.2	(66.7, 71.6)	58.1	(56.4, 59.9)	–16.0	<0.001
Making own soups, sauces, and salad dressings	57.2	(54.5, 59.8)	50.0	(48.3, 51.8)	–12.5	<0.001
Tasting food before adding salt	79.3	(77.0, 81.4)	70.5	(68.9, 72.1)	–11.0	<0.001
Draining and rinsing canned foods	57.5	(54.7, 60.2)	56.3	(54.5, 58.1)	–2.1	0.468
Food selection						
Requesting no added salt in restaurants	5.1	(4.2, 6.1)	13.7	(12.5, 15.0)	171.1	<0.001
Eating fewer packaged, ready-to-eat foods	69.3	(66.6, 71.8)	52.3	(50.6, 54.1)	–24.5	<0.001
Limiting restaurant foods	47.2	(44.5, 49.8)	36.7	(35.0, 38.4)	–22.2	<0.001
Eating more fresh fruits and vegetables	80.6	(78.4, 82.7)	67.9	(66.3, 69.6)	–15.8	<0.001
Requesting sauces/dressings on side	35.2	(32.8, 37.6)	33.6	(31.9, 35.4)	–4.4	0.298
Food labels						
Reading nutrition facts table to determine sodium content	54.2	(51.6, 56.8)	49.6	(47.9, 51.4)	–8.4	0.005
Buying low/reduced-sodium products	45.7	(43.2, 48.4)	44.6	(42.9, 46.4)	–2.5	0.475
Looking or would look for healthy choice symbol or logo	29.5	(27.3, 31.8)	46.2	(44.4, 47.9)	56.4	<0.001
Looking or would look for low-sodium message or claim	38.8	(36.4, 41.3)	49.3	(47.6, 51.1)	27.1	<0.001
Checking or would check sodium %DV on label	53.6	(51.0, 56.2)	50.9	(49.2, 52.7)	–5.0	0.094
Checking or would check sodium (mg) on label	55.7	(53.1, 58.2)	53.3	(51.6, 55.1)	–4.2	0.145

Abbreviations: CI, confidence interval; DV, daily value; RPC, relative percentage change.

Regression models revealed varying results by sex and age. Females were less likely in 2024 than in 2011 to limit restaurant foods (OR = 0.54; 95% CI: 0.45, 0.65; *P* < 0.001) or read food labels (OR = 0.71; 95% CI: 0.59, 0.85; *P* < 0.001). In contrast, requesting “no added salt” at restaurants increased among both males (OR = 3.95; 95% CI: 2.63, 5.94; *P* < 0.001) and females (OR = 2.10; 95% CI: 1.50, 2.94; *P* < 0.001). Younger participants aged 20 to 29 and 30 to 39 y were more likely to request “no added salt” at restaurants (OR = 24.62; 95% CI: 3.40, 178.08; and OR = 5.08; 95% CI: 2.25, 11.44; respectively; both *P* < 0.001). Conversely, requesting sauces on the side decreased among females (OR = 0.70; 95% CI: 0.58, 0.84; *P* < 0.001) and among participants aged 50 to 59 y (OR = 0.61; 95% CI: 0.44, 0.85; *P* = 0.001) from 2011 to 2024. Tasting food before adding salt also declined with age in 2024 (*P* = 0.002), particularly among those aged 30 to 39, 40 to 49, and 50 to 59 y (OR = 0.53; 95% CI: 0.34, 0.82; to OR = 0.54, 95% CI: 0.37, 0.81; to OR = 0.53; 95% CI: 0.37, 0.77; all *P* ≤ 0.001) (Supplemental Table 5).

### Changes in barriers to dietary sodium reduction

Several barriers to sodium reduction became more prevalent from 2011 to 2024 (Table 6), including taste concerns (mean score = 2.7–3.2; mean difference = 0.5; 95% CI: 0.4, 0.6; *P* < 0.001; 28.9%–41.6% agreeing), lack of time to prepare lower-sodium meals from scratch (mean score = 2.5–3.1; mean difference = 0.6; 95% CI: 0.5, 0.7; *P* < 0.001; 26.6%–40.0% agreeing), higher cost of low-sodium products (mean score = 2.1–2.9; mean difference = 0.8; 95% CI: 0.7, 0.9; *P* < 0.001; 13.4%–29.2% agreeing), and lack of social support (mean score = 1.9–2.4; mean difference = 0.5; 95% CI: 0.4, 0.6; *P* < 0.001; 8.8%–21.2% agreeing). Other barriers remained relatively unchanged, including not knowing how to reduce sodium (19.1% in 2011–20.5% in 2024 agreeing), difficulty understanding sodium information on food labels (30.7%–25.1% agreeing), and limited or no lower-sodium options in restaurants; both sit-down (65.4%–63.8% agreeing) and fast food (74.0%–72.8% agreeing) (Table 6). Despite limited change, the high prevalence of these barriers indicates persistent challenges to sodium reduction such as those related to restaurant options.

**TABLE 6:** Reported barriers to sodium reduction, 2011 and 2024

	2011 (*n =* 2603)	95% CI	2024 (*n* = 3267)	95% CI	RPC/MD	*P* value
Lower-sodium foods do not taste as good as regular products						
Disagree (%)	44.0	(41.4, 46.6)	24.1	(22.6, 25.7)	–45.2	<0.001
Neutral (%)	27.1	(24.8, 29.6)	34.2	(32.6, 35.9)	26.2	
Agree (%)	28.9	(26.5, 31.3)	41.6	(39.9, 43.4)	44.0	
Mean score	2.7	(2.7, 2.8)	3.2	(3.2, 3.3)	0.5 (0.4, 0.6)	<0.001
Price difference between low-sodium and regular foods is too high						
Disagree (%)	65.0	(62.3, 67.5)	31.2	(29.5, 32.9)	–52.0	<0.001
Neutral (%)	21.6	(19.4, 24.0)	39.6	(37.8, 41.4)	83.3	
Agree (%)	13.4	(11.7, 15.4)	29.2	(27.6, 30.9)	117.9	
Mean score	2.1	(2.1, 2.2)	2.9	(2.9, 3.0)	0.8 (0.7, 0.9)	<0.001
Lack of time to prepare lower-sodium meals from scratch						
Disagree (%)	57.5	(54.8, 60.1)	31.5	(29.9, 33.2)	–45.2	<0.001
Neutral (%)	15.9	(14.0, 18.0)	28.5	(27.0, 30.2)	79.2	
Agree (%)	26.6	(24.2, 29.1)	40.0	(38.2, 41.7)	50.4	
Mean score	2.5	(2.4, 2.5)	3.1	(3.0, 3.1)	0.6 (0.5, 0.7)	<0.001
Lack of support from family/relatives/friends						
Disagree (%)	76.5	(74.0, 78.9)	54.0	(52.1, 55.9)	–29.4	<0.001
Neutral (%)	14.7	(12.8, 16.8)	24.7	(23.2, 26.4)	68.0	
Agree (%)	8.8	(7.2, 10.7)	21.2	(19.8, 22.8)	140.9	
Mean score	1.9	(1.8, 1.9)	2.4	(2.3, 2.4)	0.5 (0.4, 0.6)	<0.001
Limited or no low-sodium options at fast food restaurants						
Disagree (%)	10.4	(8.7, 12.4)	7.1	(6.2, 8.1)	–31.7	<0.001
Neutral (%)	15.7	(13.7, 17.9)	20.1	(18.7, 21.6)	28.0	
Agree (%)	74.0	(71.3, 76.4)	72.8	(71.2, 74.4)	–1.6	
Mean score	4.0	(4.0, 4.1)	4.0	(4.0, 4.0)	0.0 (-0.1, 0.0)	0.478
Limited or no low-sodium options at sit-down restaurants						
Disagree (%)	13.2	(11.4, 15.2)	9.6	(8.6, 10.8)	–27.3	<0.001
Neutral (%)	21.4	(19.1, 23.8)	26.5	(25.0, 28.2)	23.8	
Agree (%)	65.4	(62.7, 68.0)	63.8	(62.1, 65.6)	–2.4	
Mean score	3.8	(3.8, 3.9)	3.8	(3.7, 3.8)	0.0 (-0.1, 0.0)	0.283
Do not know how to reduce dietary sodium						
Disagree (%)	58.2	(55.5, 60.8)	56.1	(54.4, 57.9)	–3.6	0.419
Neutral (%)	22.7	(20.5, 25.0)	23.4	(22.0, 24.9)	3.1	
Agree (%)	19.1	(17.1, 21.4)	20.5	(19.1, 21.9)	7.3	
Mean score	2.4	(2.3, 2.4)	2.4	(2.4, 2.5)	0.1 (0.0, 0.1)	0.156
Difficult to understand sodium information on food labels						
Disagree (%)	48.6	(45.9, 51.2)	47.0	(45.2, 48.7)	–3.3	<0.001
Neutral (%)	20.7	(18.7, 22.8)	27.9	(26.4, 29.5)	34.8	
Agree (%)	30.7	(28.4, 33.2)	25.1	(23.6, 26.6)	–18.3	
Mean score	2.7	(2.6, 2.8)	2.6	(2.6, 2.7)	–0.1 (–0.2, 0.0)	0.024

Abbreviations: CI, confidence interval; MD, mean difference; RPC, relative percentage change.

Age-stratified analyses showed that perceived lack of time to prepare lower-sodium meals from scratch increased most among younger participants from 2011 to 2024 (20–29 and 30–39 y; $\hat{\beta }$ = 0.78; 95% CI: 0.60, 0.97; to $\hat{\beta }$ = 0.82; 95% CI: 0.56, 1.08; *P* < 0.001) and least among older participants (60–69 y; $\hat{\beta }$ = 0.33; 95% CI: 0.33, 0.50; *P* < 0.001). Furthermore, not knowing how to reduce sodium increased among the youngest (20–29 y; $\hat{\beta }$ = 0.63; 95% CI: 0.40, 0.87; *P* < 0.001) but decreased among the oldest participants (60–69 y; $\hat{\beta }$ = –0.28; 95% CI: –0.42, –0.14; *P* = 0.001) from 2011 to 2024. Likewise, difficulty understanding sodium information on food labels increased among young (20–29 y; $\hat{\beta }$ = 0.51; 95% CI: 0.28, 0.74; *P* < 0.001) but decreased among older participants (50–59 and 60–69 y; $\hat{\beta }$ = –0.34; 95% CI: –0.49, –0.19; and $\hat{\beta }$ = –0.39; 95% CI: –0.55, –0.23; *P* < 0.001) (Supplemental Table 6).

## Discussion

This study provides the first national assessment of temporal changes in dietary sodium KAB among Canadian adults using repeated cross-sectional data from 2011 and 2024. Contrary to our hypothesis, most indices of population-level sodium KAB deteriorated over time. Despite persistently high concern about excess sodium intake, fewer Canadians reported taking action to reduce sodium in 2024, including avoiding high-sodium foods, limiting added salt, and choosing fresh produce. Additionally, only modest improvements were observed in the knowledge and skills needed to reduce dietary sodium (e.g., attention to front-of-package symbols and claims). Barriers to sodium reduction, particularly cost, taste, time constraints, and limited social or healthcare provider support, became more prevalent, with younger adults and males reporting greater difficulty reducing sodium intake and interpreting labels. Motivation to reduce sodium increasingly reflected the management of health conditions and encouragement from healthcare providers, whereas a lack of healthcare provider guidance emerged as a common reason for inaction. Concerningly, knowledge of the association between excess dietary sodium and major cardiometabolic outcomes decreased over time, and several misconceptions persisted.

This study makes a novel contribution by documenting population-level trends in sodium KAB over time. Despite excess sodium intake being a longstanding global public health priority, and WHO’s recommendations for ongoing surveillance of sodium intake and related behavioral indicators [24,30], routine monitoring of population-level sodium KAB remains limited [31], with most assessments conducted as part of predominantly clinically-focused sodium-reduction interventions [[32], [33], [34]]. One of the only known studies that also assessed national-level temporal changes in dietary sodium KAB was conducted among American adults (2012 and 2015, *n =* 7796). This study showed modest improvements in sodium knowledge and behaviors, which could be attributed to sustained public education campaigns emphasizing processed and restaurant foods as major sodium sources [28]. In contrast, our findings show less favorable trends in Canada, with a decline in sodium-related health knowledge, and widespread reductions in behavioral engagement. Attitudinal patterns also differed: although there was little change in the extent that American adults perceive harm associated with excess dietary sodium, Canadian participants had an increase in perceived harmfulness but paradoxically an unchanged concern about excess sodium. In the absence of a dedicated sodium-specific surveillance system, this study provides rare and timely national insights [35].

The discrepancy between the declining sodium-related KAB observed in our study, and the relatively stable national-level sodium intake estimates (after accounting for underreporting) [10,21], should be interpreted in light of important national data limitations. Canada’s most recent nationally representative sodium intake data were collected in 2015, almost 10 y before the 2024 data collection in this study, limiting direct comparison with 2024 KAB findings [10]. Stable intake estimates may also mask opposing influences. Modest or absent changes in individual sodium-reduction behaviors are counterbalanced by continued high exposure to sodium from processed and restaurant foods, resulting in little net change in average intake. As such, sodium KAB indicators should be viewed as complementary, upstream measures of behavioral capacity and opportunity, rather than direct proxies for population sodium intake. Future research should conduct direct comparisons between dietary sodium KAB and dietary sodium intakes at the national level.

The findings of this study should be interpreted cautiously given the repeated cross-sectional design and the potential unmeasured contextual influences. Nevertheless, they are broadly consistent with evidence that Canada has made comparatively slow progress in implementing comprehensive sodium-reduction measures [8,36]. Although mandatory “high in” FOPL are scheduled for implementation in Canada in 2026, Canada’s voluntary sodium reformulation targets for packaged foods have shown limited effectiveness over the past decade [15,[37], [38], [39]]. Sodium reduction has also tended to be included in broader healthy eating initiatives, rather than prioritized as a distinct public health focus, unlike tobacco or physical inactivity [17,18,[40], [41], [42]], an issue highlighted in qualitative research with Canadians [43]. At the same time, several nonpolicy drivers may contribute to the observed trends, including competing nutrition messages (e.g., emphasis on sugar reduction, weight management), inflation and economic pressures affecting food affordability and food security, generational differences in health information sources, and increased exposure to nutrition misinformation and disinformation through digital media [[44], [45], [46], [47]]. The COVID-19 pandemic may have further shifted food purchasing and preparation practices and may have reduced access to healthcare advice [44]. In contrast, countries that integrated sodium-reduction considerations within broader nutrition policy approaches (e.g., warning labels, marketing restrictions, fiscal measures) have shown greater progress by creating more supportive environments for sodium reduction [8,48]. Several cognitive and informational challenges may further explain our findings. Declining label use, despite simplified % DV interpretations [49], and weakening knowledge of sodium-related health risks may also reflect both the cognitive burden of navigating high-sodium food environments and the influence of a fragmented digital information landscape. This challenge may be amplified by the difficulty of monitoring sodium that is “hidden” in commonly consumed foods not perceived as salty (e.g., bread, sauces) [[50], [51], [52]] and by optimistic self-assessment of sodium intake, which may reduce the perceived urgency to act [53,54].

Collectively, our findings underscore that concern, or knowledge alone is insufficient without supportive policies and environments that support healthier and lower-sodium choices. For instance, Canada’s new front-of-package labeling regulations, nationally launching in January 2026, represent a strategic opportunity to increase awareness of hidden sodium and shift social norms. These “High In” warning labels will identify products high in sodium, saturated fat and sugar [16]. However, their effectiveness will likely depend on complementary measures including sodium-focused public communication, food literacy initiatives, and healthcare provider engagement, particularly for younger adults and males, who consistently showed lower engagement [16,55,56]. Ongoing sodium KAB surveillance will also be essential to monitor implementation impacts, identifying emerging barriers, and guide equitable, population-specific strategies [24,32,33].

### Strengths and limitations

This study has several strengths, including the use of large, nationally weighted surveys, the use of validated survey instruments administered at 2 time points, and its novelty and ability to inform public health policy related to dietary sodium reduction. The substantial sample sizes provided sufficient statistical power to detect meaningful shifts in sodium KAB across demographic groups, and interpretation emphasized both statistical and practical significance [29]. Expert and stakeholder input enhanced policy relevance and consistency of measures over time. Nonetheless, several limitations warrant consideration. First, independent cross-sectional samples recruited from different online panels (Advanced Foods and Materials Network [AFMN] in 2011 and Leger panel in 2024) may introduce unmeasured selection bias, despite weighting both samples to national census benchmarks. Some socioeconomic variables were not collected in 2011, limiting full cohort comparability. Second, repeated cross-sectional comparisons cannot establish causality and may reflect both true population change and cohort or sampling variation. Third, although the exclusion of specific response types (“I do not know” and item nonresponse*)* may have affected descriptive distributions, sensitivity analyses using multiple imputation yielded results consistent with the complete-case analysis in both direction and magnitude, supporting the robustness of the findings. Fourth, despite Bonferroni-adjusted post hoc testing and a conservative significance threshold, the potential for residual type I error cannot be fully excluded. Finally, self-reported data are subject to recall and social desirability bias, although anonymity and voluntary participation may have mitigated these effects.

In summary, over a 13-y period, multiple indicators of sodium KAB deteriorated among Canadian adults, with only few improvements observed. These findings highlight a widening consumer knowledge-to-action gap and reinforce the need for comprehensive public health efforts to support population-wide dietary sodium reduction. Such efforts include strengthened food environments to improve availability and access to lower-sodium foods, enhanced sodium-focused communication and education, and greater engagement of public health and healthcare professionals to mitigate the ongoing burden of dietary sodium-related chronic disease.

## Author contributions

The authors’ responsibilities were as follows – JA, MLA, WL, RAG: designed the research; RAG, JA: oversaw data collection; RAG, MP: analyzed the data; RAG: wrote the manuscript; JA: has primary responsibility for all aspects of the study; and all authors: revised the manuscript, read and approved the final manuscript.

## Data availability

Data described in the manuscript will be made available on request pending the corresponding author’s approval.

## Declaration of artificial intelligence

AI was not used in this submission.

## Funding

This work was supported by the Canadian Institutes of Health Research (#PJT187686, J. Arcand). RAG is funded by a Canada Graduate Scholarship – Doctoral Award (#RN497185-493397).

## Conflict of interest

JA and RAG report a relationship with Canadian Institute of Health Research that includes: funding grants. Other authors declare that they have no known competing financial interests or personal relationships that could have appeared to influence the work reported in this paper.
