# Supplementary material for: Changes in dietary sodium knowledge, attitudes and behaviors among Canadian adults in 2011 and 2024: a repeated cross-sectional study
Source: Am J Clin Nutr. 2026 Jan 27;123(4):101213. doi: 10.1016/j.ajcnut.2026.101213 (PMC13084583; doi:10.1016/j.ajcnut.2026.101213)
Supplement: multimedia component 1 [file mmc1.docx]

**Supplementary Material**

**Supplementary Appendix 1. Common sodium-related knowledge, attitude, and behaviour survey questions used in 2011 and 2024**

Please refer to the following paper for questionnaire validation: Al Ghali R, Prashad M, Lou W, Arcand J. Development and Validation of an Instrument to Assess the Cognitive, Behavioral, and Environmental Factors Related to Sodium Intake in Adult Canadians: The Behavioral Assessment Instrument for Dietary Sodium. *Curr Dev Nutr*. 2025;9(11):107592. doi:10.1016/j.cdnut.2025.107592. Available from: [https://www.sciencedirect.com/science/article/pii/S2475299125030549](https://www.sciencedirect.com/science/article/pii/S2475299125030549?utm_source=chatgpt.com).

1. Using the following scale, how would you rate your overall health? (*Check one*)

| Very Poor Very Good | | | | |
| --- | --- | --- | --- | --- |
| 1 | 2 | 3 | 4 | 5 |
|  |  |  |  |  |

1. Overall, how healthy do you consider your diet to be? (*Check one*)

| Very Unhealthy Very Healthy | | | | |
| --- | --- | --- | --- | --- |
| 1 | 2 | 3 | 4 | 5 |
|  |  |  |  |  |

1. To the best of your knowledge, how much do you think sodium affects your overall health? *(Check one)*

| Very little A great deal | | | | | I do not know |
| --- | --- | --- | --- | --- | --- |
| (1) | (2) | (3) | (4) | (5) |  |
|  |  |  |  |  |  |

1. To the best of your knowledge, which of the following medical conditions are associated or are not associated with high sodium intake? *(Check one per line)*

|  | Associated | Not associated | I do not know |
| --- | --- | --- | --- |
| a. High Blood Pressure |  |  |  |
| b. Osteoporosis |  |  |  |
| c. Arthritis |  |  |  |
| d. Depression |  |  |  |
| e. Heart Disease |  |  |  |
| f. Stroke |  |  |  |
| g. Diabetes |  |  |  |

1. To the best of your knowledge, what is the maximum amount of sodium that adults should have in one day? *(Check one)*

| a. 900 mg |  |
| --- | --- |
| b. 1200 mg |  |
| c. 1500 mg |  |
| d. 2000 mg |  |
| e. 2300 mg |  |
| f. 2800 mg |  |
| g. 3400 mg |  |
| h. I do not know |  |

*Text reads:* “There are a number of brands of crackers in Canada that range from low to high sodium. On the next screen, we will show you a Nutrition Facts table from one of these boxes of crackers.” Subjects to be randomly assigned one of the following low, medium, or high Nutrition Facts tables.

1. This is a Nutrition Facts table for a box of crackers. If we were to ask you to judge how much sodium is in this product, would you say it is low, medium, or high sodium? *(Check one)*


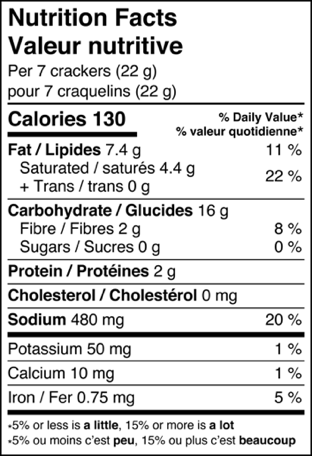

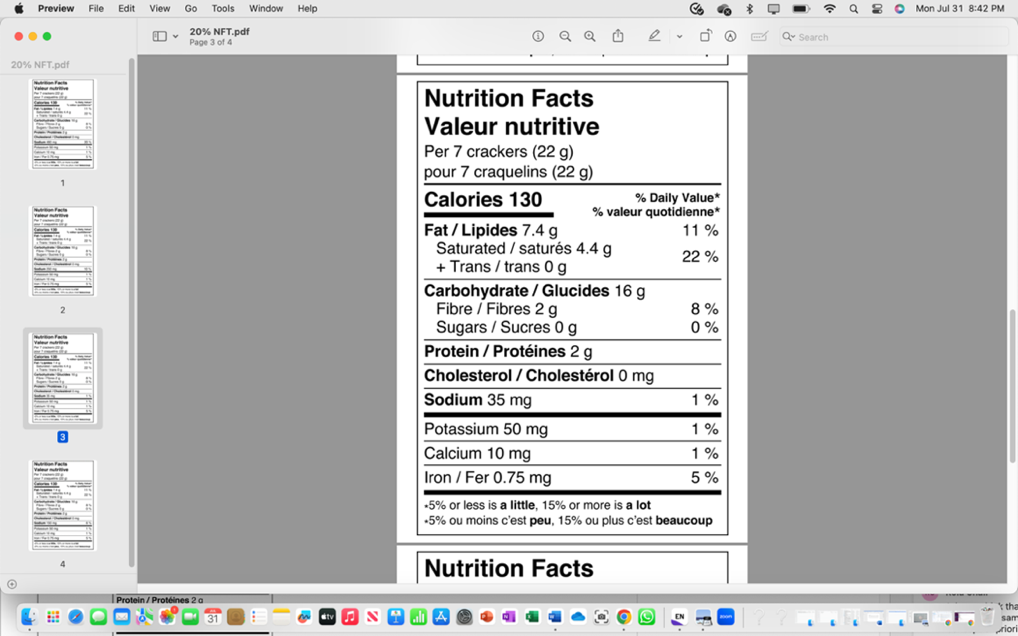

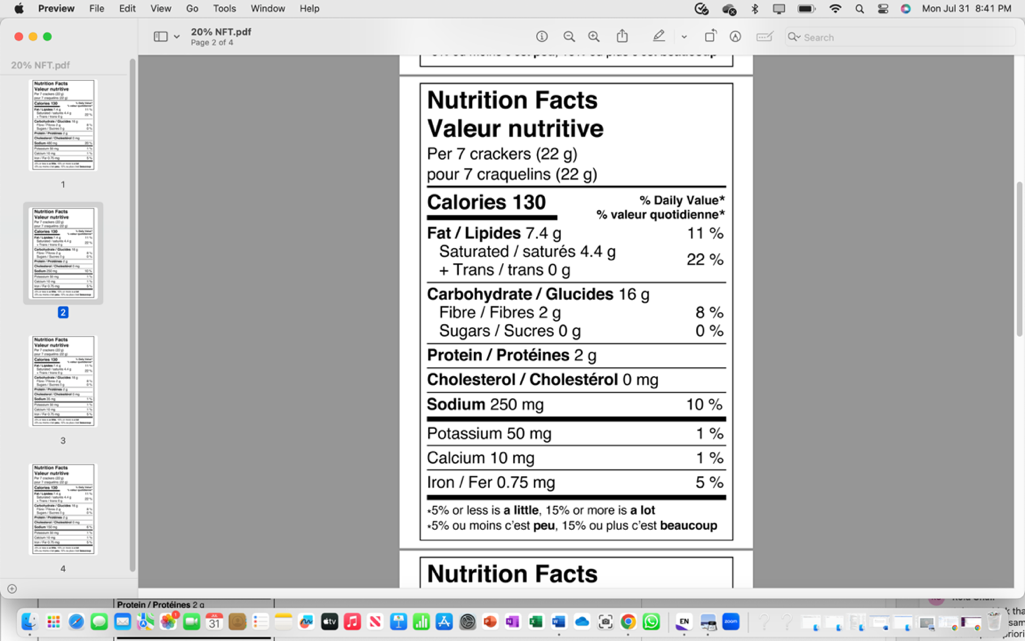


1. Low a. Low a. Low
2. Medium b. Medium b. Medium
3. High c. High c. High
4. I do not know d. I do not know d. I do not know
5. Please tell us to what extent you agree or disagree with the following statements. *(Check one per line)*

|  | Strongly Strongly  Disagree Agree | | | | |
| --- | --- | --- | --- | --- | --- |
|  | 1 | 2 | 3 | 4 | 5 |
| a. Kosher salt, sea salt and gourmet salts contain less sodium than regular table salt |  |  |  |  |  |
| b. My sodium intake is low because I do not add salt to my food |  |  |  |  |  |
| c. People with low or normal blood pressure do not need to be concerned with the amount of sodium in their diet |  |  |  |  |  |

1. Which of the following best describes your approach towards sodium? *(Check one)*
2. I am not limiting my sodium intake. (*Skip to Q10)*
3. I am interested in limiting my sodium intake, but have not yet started. (*Go to Q9*)
4. I have tried limiting my sodium intake in the past, but not anymore. (*Go to Q9*)
5. I am currently trying to limit my sodium intake. (*Go to Q9*)

Only asked to those LIMITING sodium, based on Q8: “I am interested in limiting my sodium intake”, “I have tried limiting my sodium intake but not anymore”, “I am currently trying to limit my sodium intake”

1. Please tell us the extent to which you agree or disagree with the following statements as reasons for limiting your sodium intake. *(Check one per line)*

|  | Strongly Strongly  Disagree Agree | | | | | Not Applicable |
| --- | --- | --- | --- | --- | --- | --- |
|  | 1 | 2 | 3 | 4 | 5 |  |
| 1. To manage a current health condition |  |  |  |  |  |  |
| b. Because my health care provider has recommended it |  |  |  |  |  |  |

Only asked to those NOT LIMITING, based on Q8: “I am not limiting my sodium intake”.

1. Please tell us the extent to which you agree or disagree with the following statements as reasons for not limiting your sodium intake. *(Check one per line)*

|  | Strongly Strongly  Disagree Agree | | | | | Not Applicable |
| --- | --- | --- | --- | --- | --- | --- |
|  | 1 | 2 | 3 | 4 | 5 |  |
| a. Sodium is not bad for me |  |  |  |  |  |  |
| b. There is conflicting information about whether I should or should not reduce my sodium |  |  |  |  |  |  |
| c. I have a normal or low blood pressure |  |  |  |  |  |  |
| d. I do not need to limit my sodium since I am on blood pressure medication |  |  |  |  |  |  |
| e. My health care provider has not recommended it |  |  |  |  |  |  |

1. Over the past month, can you think of an occasion when you decided to avoid purchasing or consuming a particular food because you thought it was too high in sodium? *(Check one)*
2. Yes
3. No
4. I do not know/remember
5. Below we have listed some of the common ways to lower the amount of sodium in your diet. In the past month, to what extent have you personally done any of the following, regardless of whether or not you are trying to limit your sodium? (*Check one per line*)

|  | Never Always  do this do this | | | | | Not Applicable |
| --- | --- | --- | --- | --- | --- | --- |
|  | 1 | 2 | 3 | 4 | 5 |  |
| a. Avoid adding salt during cooking |  |  |  |  |  |  |
| b. Avoid adding salt at the table |  |  |  |  |  |  |
| c. Make your own soups, sauces, and salad dressings |  |  |  |  |  |  |
| d. Eat more fresh fruits and vegetables |  |  |  |  |  |  |
| e. Eat fewer packaged, ready-to-eat foods |  |  |  |  |  |  |
| f. Taste your food before adding salt |  |  |  |  |  |  |
| g. Use spices, herbs and/or seasonings instead of salt during cooking |  |  |  |  |  |  |
| - 1. Drain and rinse canned vegetables and beans/legumes before use |  |  |  |  |  |  |
| i. Limit or avoid eating food made in restaurants or cafeterias |  |  |  |  |  |  |
| j. At restaurants, ask to have your meal prepared without salt |  |  |  |  |  |  |
| k. At restaurants, ask for dressings and sauces on the side |  |  |  |  |  |  |
| l. Read the Nutrition Facts table to determine if a product is high or low sodium |  |  |  |  |  |  |
| m. Buy foods labelled as “low sodium” or “reduced sodium” or “sodium free”, when available |  |  |  |  |  |  |

1. When there are many products to choose from, we are interested to know how you would find the lowest sodium choice. To what extent do you/would you do the following in order to find the lowest sodium product? *(Check one per line)*

|  | Never Always  do this do this | | | | |
| --- | --- | --- | --- | --- | --- |
|  | 1 | 2 | 3 | 4 | 5 |
| a. Look at the total amount of sodium (mg) on the Nutrition Facts table |  |  |  |  |  |
| b. Look at the % Daily Value (% DV) on the Nutrition Facts table |  |  |  |  |  |
| c. Look for a symbol or a logo on the food package that suggests a product is a healthy choice |  |  |  |  |  |
| d. Look for a message or claim on the food package that says a product is “low sodium”, “reduced sodium” or “sodium free” |  |  |  |  |  |

1. How much sodium do you think you consume? *(Check one)*
2. Far too much
3. Too much
4. The right amount
5. Too little
6. Far too little
7. I do not know
8. How do you think your own intake of sodium compares with the average Canadian adult? (*Check one*)
   - - - 1. Much lower
         2. Somewhat lower
         3. About the same
         4. Somewhat higher
         5. Much higher
         6. I do not know
9. Please tell us the extent to which you agree or disagree with the following statement. *(Check one per line)*

|  | Strongly Strongly  Disagree Agree | | | | |
| --- | --- | --- | --- | --- | --- |
|  | 1 | 2 | 3 | 4 | 5 |
| My health would improve if I lowered the amount of sodium I eat |  |  |  |  |  |

1. How concerned are you about the amount of sodium in your diet? *(Check one)*
2. Extremely concerned
3. Very concerned
4. Somewhat concerned
5. Not very concerned
6. Not at all concerned
7. Please tell us the extent to which you agree or disagree with the following statement. *(Check one per line)*

|  | Strongly Strongly  Disagree Agree | | | | | Not Applicable |
| --- | --- | --- | --- | --- | --- | --- |
|  | 1 | 2 | 3 | 4 | 5 |  |
| In general, lower sodium food products do not taste as good, compared to regular products |  |  |  |  |  |  |

1. Please tell us the extent to which you agree or disagree with the following statements, regardless of whether or not you are trying to limit your sodium intake. *(Check one per line)*

|  | Strongly Strongly  Disagree Agree | | | | | Not Applicable |
| --- | --- | --- | --- | --- | --- | --- |
|  | 1 | 2 | 3 | 4 | 5 |  |
| a. The price difference between low-sodium and regular foods is too high for me |  |  |  |  |  |  |
| b. I do not always have time to prepare lower sodium meals from scratch |  |  |  |  |  |  |

1. Several social factors may affect lowering the amount of sodium in your diet. Please tell us the extent to which you agree or disagree with the following statement, regardless of whether or not you are trying to limit your sodium intake. *(Check one per line)*

|  | Strongly Strongly  Disagree Agree | | | | | Not Applicable |
| --- | --- | --- | --- | --- | --- | --- |
|  | 1 | 2 | 3 | 4 | 5 |  |
| A lack of support from my family members, relatives and/or friends makes reducing sodium difficult |  |  |  |  |  |  |

1. Several environmental factors may affect lowering the amount of sodium in your diet. Please tell us the extent to which you agree or disagree with the following statements, regardless of whether or not you are trying to limit your sodium intake. *(Check one per line)*

|  | Strongly Strongly  Disagree Agree | | | | | Not Applicable |
| --- | --- | --- | --- | --- | --- | --- |
|  | 1 | 2 | 3 | 4 | 5 |  |
| a. When eating at fast food restaurants, I find that lower sodium options are not available or only in limited variety |  |  |  |  |  |  |
| b. When eating at sit-down restaurants, I find that lower sodium options are not available or only in limited variety |  |  |  |  |  |  |

1. Please tell us the extent to which you agree or disagree with the following statements, regardless of whether or not you are trying to limit your sodium intake. *(Check one per line)*

|  | Strongly Strongly  Disagree Agree | | | | | Not Applicable |
| --- | --- | --- | --- | --- | --- | --- |
|  | 1 | 2 | 3 | 4 | 5 |  |
| a. I do not know how to reduce the amount of sodium I eat |  |  |  |  |  |  |
| b. It is difficult to understand sodium information on food labels |  |  |  |  |  |  |

1. Are you a parent or caregiver for a child/children under 18 years of age who lives in your household? *(Check one)*
2. Yes *(Go to Q40)*
3. No *(Skip to Q44)*
4. Are you concerned about the sodium intake of the child/children in your household? *(Check one)*
5. Yes
6. No

**Supplementary Table 1. Item-level missing data patterns (‘do not know’ and nonresponse) for sodium-related variables in 2011 and 2024.**

| **Question statement** | **Number and type of missingness in 2011 (n=2603)** | **%** | **Number and type of missingness in 2024**  **(n=3267)** | **%** |
| --- | --- | --- | --- | --- |
| Using the following scale, how would you rate your overall health? (*Check one*) | 13 NR | 0.5 | 0 | 0.0 |
| Overall, how healthy do you consider your diet to be? (*Check one*) | 12 NR | 0.5 | 0 | 0.0 |
| To the best of your knowledge, how much do you think sodium affects your overall health? *(Check one)* | 0 | 0.0 | 0 | 0.0 |
| Association between sodium and high blood pressure | 24 NR | 0.9 | 0 | 0.0 |
| Association between sodium and osteoporosis | 73 NR | 2.8 | 0 | 0.0 |
| Association between sodium and arthritis | 82 NR | 3.1 | 0 | 0.0 |
| Association between sodium and depression | 125 NR | 4.8 | 0 | 0.0 |
| Association between sodium and heart disease | 41 NR | 1.6 | 0 | 0.0 |
| Association between sodium and stroke | 51 NR | 2.0 | 0 | 0.0 |
| Association between sodium and diabetes | 63 NR | 2.4 | 0 | 0.0 |
| To the best of your knowledge, what is the maximum amount of sodium that adults should have in one day? *(Check one)* | 6 NR | 0.2 | 0 | 0.0 |
| This is a Nutrition Facts table for a box of crackers. If we were to ask you to judge how much sodium is in this product, would you say it is low, medium, or high sodium? *(Check one)* | 67 NR | 2.6 | 0 | 0.0 |
| Kosher salt, sea salt and gourmet salts contain less sodium than regular table salt | 18 NR | 0.7 | 0 | 0.0 |
| My sodium intake is low because I do not add salt to my food | 12 NR | 0.5 | 0 | 0.0 |
| People with low or normal blood pressure do not need to be concerned with the amount of sodium in their diet | 18 NR | 0.7 | 0 | 0.0 |
| Which of the following best describes your approach towards sodium? *(Check one)* | 11 NR | 0.4 | 0 | 0.0 |
| Over the past month, can you think of an occasion when you decided to avoid purchasing or consuming a particular food because you thought it was too high in sodium? *(Check one)* | 139 NR  110 DNK | 5.3  4.2 | 372 DNK | 11.4 |
| Avoid adding salt during cooking | 33 NR | 1.3 | 0 | 0.0 |
| Avoid adding salt at the table | 59 NR | 2.3 | 0 | 0.0 |
| Make your own soups, sauces, and salad dressings | 39 NR | 1.5 | 0 | 0.0 |
| Eat more fresh fruits and vegetables | 43 NR | 1.6 | 0 | 0.0 |
| Eat fewer packaged, ready-to-eat foods | 35 NR | 1.3 | 0 | 0.0 |
| Taste your food before adding salt | 43 NR | 1.6 | 0 | 0.0 |
| Use spices, herbs and/or seasonings instead of salt during cooking | 50 NR | 1.9 | 0 | 0.0 |
| Drain and rinse canned vegetables and beans/legumes before use | 48 NR | 1.8 | 0 | 0.0 |
| Limit or avoid eating food made in restaurants or cafeterias | 75 NR | 2.9 | 0 | 0.0 |
| At restaurants, ask to have your meal prepared without salt | 51 NR | 1.9 | 0 | 0.0 |
| At restaurants, ask for dressings and sauces on the side | 46 NR | 1.8 | 0 | 0.0 |
| Read the Nutrition Facts table to determine if a product is high or low sodium | 48 NR | 1.8 | 0 | 0.0 |
| Buy foods labelled as “low sodium” or “reduced sodium” or “sodium free”, when available | 107 NR | 4.1 | 0 | 0.0 |
| Look at the total amount of sodium (mg) on the Nutrition Facts table | 45 NR | 1.7 | 0 | 0.0 |
| Look at the % Daily Value (% DV) on the Nutrition Facts table | 50 NR | 1.9 | 0 | 0.0 |
| Look for a symbol or a logo on the food package that suggests a product is a healthy choice | 43 NR | 1.6 | 0 | 0.0 |
| Look for a message or claim on the food package that says a product is “low sodium”, “reduced sodium” or “sodium free” | 49 NR | 1.9 | 0 | 0.0 |
| How much sodium do you think you consume? *(Check one)* | 7 NR  130 DNK | 0.3  5.0 | 223 DNK | 6.8 |
| How do you think your own intake of sodium compares with the average Canadian adult? (*Check one*) | 81 DNK | 3.1 | 220 DNK | 6.7 |
| My health would improve if I lowered the amount of sodium I eat | 19 NR | 0.7 | 0 | 0.0 |
| How concerned are you about the amount of sodium in your diet? *(Check one)* | 5 NR | 0.2 | 0 | 0.0 |
| In general, lower sodium food products do not taste as good, compared to regular products | 26 NR | 1.0 | 0 | 0.0 |
| The price difference between low-sodium and regular foods is too high for me | 62 NR | 2.4 | 0 | 0.0 |
| I do not always have time to prepare lower sodium meals from scratch | 48 NR | 1.9 | 0 | 0.0 |
| A lack of support from my family members, relatives and/or friends makes reducing sodium difficult | 53 NR | 2.0 | 0 | 0.0 |
| When eating at fast food restaurants, I find that lower sodium options are not available or only in limited variety | 51 NR | 1.9 | 0 | 0.0 |
| When eating at sit-down restaurants, I find that lower sodium options are not available or only in limited variety | 48 NR | 1.9 | 0 | 0.0 |
| I do not know how to reduce the amount of sodium I eat | 62 NR | 2.3 | 0 | 0.0 |
| It is difficult to understand sodium information on food labels | 52 NR | 2.0 | 0 | 0.0 |

NR: Nonresponse, DKN: **Do not know**

**Supplementary Table 2. Action and reasons for limiting or not limiting dietary sodium: Changes and group-year interactions, 2011 and 2024.**

|  | **2011 (n=2,603)** | | **2024 (n=3,267)** | | |  | | | |  |  |  |  |  |  |
| --- | --- | --- | --- | --- | --- | --- | --- | --- | --- | --- | --- | --- | --- | --- | --- |
|  | **%/MS** | **95% CI** | **%/MS** | | **95% CI** | | **RPC/MD** | | ***P* value** | | **Group-Year Interaction *P* value** | **OR/** **Linear regression (**$\hat{\boldsymbol{\beta}}$**)** | **95% CI** | **Interaction**  ***P* value** |  |
| Taking action to reduce sodium | | | | | | | | | | |  |  |  |  |  |
| Overall (%) | 57.4 | (54.8, 60.0) | 37.3 | (35.6, 39.0) | | -35.1 | | <0.001 | | |  |  |  |  |  |
| Sex (%) | | | | | | | | | | | 0.297 |  |  |  |  |
| Female | 57.3 | (54.1, 60.4) | 38.73 | (36.4, 41.1) | | -32.4 | | <0.001 | | |  |  |  |  |  |
| Male | 57.49 | (53.2, 61.6) | 35.69 | (33.3, 38.1) | | -37.9 | | <0.001 | | |  |  |  |  |  |
| Age group (years) (%) | | | | | | | | | | | 0.002 |  |  |  |  |
| 20-29 | 37.6 | (29.6, 46.2) | 14.6 | (12.0, 17.7) | | -61.0 | | <0.001 | | |  | 0.28 | (0.16, 0.50) | <0.001 |  |
| 30-39 | 53.1 | (47.4, 58.8) | 25.1 | (22.1, 28.4) | | -52.7 | | <0.001 | | |  | 0.30 | (0.20, 0.43) | <0.001 |  |
| 40-49 | 58.9 | (54.5, 63.2) | 33.4 | (29.8, 37.3) | | -43.2 | | <0.001 | | |  | 0.35 | (0.25, 0.48) | <0.001 |  |
| 50-59 | 68.0 | (64.0, 71.8) | 49.9 | (45.8, 54.0) | | -26.6 | | <0.001 | | |  | 0.47 | (0.34, 0.64) | <0.001 |  |
| 60-69 | 72.5 | (67.9, 76.7) | 60.8 | (57.0, 64.5) | | -16.2 | | <0.001 | | |  | 0.59 | (0.41, 0.84) | 0.001 |  |
| *Reasons for limiting sodium* | | | | | | | | | | |  |  |  |  |  |
| Managing a current health condition | | | | | | | | | | |  |  |  |  |  |
| Overall |  |  |  |  | |  | |  | | |  |  |  |  |  |
| Disagree (%) | 35.7 | (32.5, 38.9) | 20.1 | (18.3, 22.0) | | –43.7 | |  | | |  |  |  |  |  |
| Neutral (%) | 18.4 | (16.0, 21.1) | 23.6 | (21.7, 25.6) | | 28.3 | | <0.001 | | |  |  |  |  |  |
| Agree (%) | 45.9 | (42.8, 49.1) | 56.4 | (54.1, 58.6) | | 22.9 | |  | | |  |  |  |  |  |
| Mean score | 3.2 | (3.1, 3.3) | 3.5 | (3.4, 3.5) | | 0.3 (0.2, 0.4) | | <0.001 | | |  |  |  |  |  |
| Sex (Mean score) | | | | | | | | | | |  |  |  |  |  |
| Female | 3.1 | (3.0, 3.2) | 3.4 | (3.3, 3.5) | | 0.3 (0.2, 0.5) | | <0.001 | | | 0.852 |  |  |  |  |
| Male | 3.2 | (3.0, 3.4) | 3.5 | (3.5, 3.6) | | 0.3 (0.1, 0.5) | | <0.001 | | |  |  |  |  |  |
| Age group (years) (Mean score) | | | | | | | | | | | 0.004 |  |  |  |  |
| 20-29 | 2.5 | (2.1, 2.9) | 3.5 | (3.3, 3.6) | | 1.0 (0.5, 1.4) | | <0.001 | | |  | 0.96 | (0.55, 1.36) | <0.001 |  |
| 30-39 | 2.9 | (2.7, 3.2) | 3.3 | (3.1, 3.4) | | 0.3 (0.1, 0.6) | | 0.012 | | |  |  |  |  |  |
| 40-49 | 3.3 | (3.2, 3.5) | 3.4 | (3.3, 3.6) | | 0.1 (-0.1, 0.3) | | 0.275 | | |  |  |  |  |  |
| 50-59 | 3.4 | (3.2, 3.5) | 3.5 | (3.4, 3.6) | | 0.1 (-0.1, 0.3) | | 0.200 | | |  |  |  |  |  |
| 60-69 | 3.5 | (3.3, 3.7) | 3.6 | (3.5, 3.8) | | 0.1 (0.0, 0.3) | | 0.136 | | |  |  |  |  |  |
| Recommended by healthcare provider | | | | | | | | | | | |  |  |  |  |
| Overall |  |  |  |  | |  | |  | | |  |  |  |  |  |
| Disagree (%) | 49.5 | (46.3, 52.6) | 36.2 | (33.9, 38.5) | | –26.9 | |  | | |  |  |  |  |  |
| Neutral (%) | 19.3 | (17.1, 21.8) | 24 | (22.0, 26.1) | | 24.4 | | <0.001 | | |  |  |  |  |  |
| Agree (%) | 31.2 | (28.5, 34.1) | 39.8 | (37.5, 42.1) | | 27.6 | |  | | |  |  |  |  |  |
| Mean score | 2.7 | (2.6, 2.8) | 3.0 | (2.9, 3.0) | | 0.3 (0.1, 0.4) | | <0.001 | | |  |  |  |  |  |
| Sex (Mean score) | | | | | | | | | | | 0.001 |  |  |  |  |
| Female | 2.7 | (2.6, 2.8) | 2.7 | (2.6, 2.8) | | 0.0 (-0.1, 0.2) | | 0.514 | | |  |  |  |  |  |
| Male | 2.8 | (2.6, 2.9) | 3.2 | (3.1, 3.3) | | 0.4 (0.2, 0.6) | | <0.001 | | |  | 0.42 | (0.25, 0.59) | <0.001 |  |
| Age group (years) (Mean score) | | | | | | | | | | | 0.003 |  |  |  |  |
| 20-29 | 2.1 | (1.8, 2.4) | 3.1 | (3.0, 3.3) | | 1.0 (0.7, 1.4) | | <0.001 | | |  | 1.02 | (0.68, 1.36) | <0.001 |  |
| 30-39 | 2.5 | (2.3, 2.7) | 2.8 | (2.6, 2.9) | | 0.3 (0.1, 0.6) | | 0.011 | | |  |  |  |  |  |
| 40-49 | 2.8 | (2.6, 2.9) | 2.8 | (2.7, 3.0) | | 0.0 (-0.2, 0.2) | | 0.829 | | |  |  |  |  |  |
| 50-59 | 3.0 | (2.8, 3.1) | 3.0 | (2.9, 3.2) | | 0.1 (-0.1, 0.3) | | 0.435 | | |  |  |  |  |  |
| 60-69 | 3.2 | (3.0, 3.4) | 3.1 | (3.0, 3.2) | | -0.1 (-0.3, 0.1) | | 0.300 | | |  |  |  |  |  |
| *Reasons for not limiting sodium* | | | | | | | | | | | |  |  |  |  |
| Believing sodium is not bad to health | | | | | | | | | | | |  |  |  |  |
| Overall | | | | | | | | | | | |  |  |  |  |
| Disagree (%) | 48.7 | (43.1, 54.3) | 38.9 | (35.5, 42.4) | | –20.1 | |  | | |  |  |  |  |  |
| Neutral (%) | 30.3 | (25.4, 35.6) | 35.7 | (32.4, 39.2) | | 17.8 | | 0.014 | | |  |  |  |  |  |
| Agree (%) | 21.1 | (16.9, 25.9) | 25.4 | (22.4, 28.6) | | 20.4 | |  | | |  |  |  |  |  |
| Mean score | 2.6 | (2.5, 2.7) | 2.8 | (2.7, 2.9) | | 0.2 (0.0, 0.3) | | 0.010 | | |  |  |  |  |  |
| Sex (Mean score) | | | | | | | | | | | 0.844 |  |  |  |  |
| Female | 2.6 | (2.4, 2.7) | 2.8 | (2.7, 2.9) | | 0.2 (0.0, 0.4) | | 0.029 | | |  |  |  |  |  |
| Male | 2.7 | (2.5, 2.9) | 2.8 | (2.7, 2.9) | | 0.2 (-0.1, 0.4) | | 0.143 | | |  |  |  |  |  |
| Age group (years) (Mean score) | | | | | | | | | | | 0.083 |  |  |  |  |
| 20-29 | 2.6 | (2.3, 2.9) | 3.0 | (2.9, 3.2) | | 0.5 (0.1, 0.8) | | 0.008 | | |  |  |  |  |  |
| 30-39 | 2.6 | (2.4, 2.8) | 3.0 | (2.8, 3.1) | | 0.4 (0.1, 0.6) | | 0.010 | | |  |  |  |  |  |
| 40-49 | 2.8 | (2.6, 3.0) | 2.8 | (2.6, 3.0) | | 0.0 (-0.3, 0.3) | | 0.807 | | |  |  |  |  |  |
| 50-59 | 2.6 | (2.4, 2.8) | 2.5 | (2.3, 2.7) | | -0.1 (-0.4, 0.2) | | 0.626 | | |  |  |  |  |  |
| 60-69 | 2.4 | (2.1, 2.7) | 2.7 | (2.5, 2.9) | | 0.3 (-0.1, 0.6) | | 0.124 | | |  |  |  |  |  |
| Conflicting information about sodium and health | | | | | | | | | | | |  |  |  |  |
| Overall | | | | | | | | | | | |  |  |  |  |
| Disagree (%) | 50.2 | (44.3, 56.0) | 38.5 | (34.9, 42.3) | | –23.3 | |  | | |  |  |  |  |  |
| Neutral (%) | 32 | (26.9, 37.4) | 36.6 | (33.0, 40.3) | | 14.4 | | 0.003 | | |  |  |  |  |  |
| Agree (%) | 17.9 | (13.9, 22.8) | 24.9 | (21.8, 28.3) | | 39.1 | |  | | |  |  |  |  |  |
| Mean score | 2.5 | (2.3, 2.6) | 2.8 | (2.7, 2.9) | | 0.3 (0.1, 0.5) | | 0.001 | | |  |  |  |  |  |
| Sex (Mean score) | | | | | | | | | | | 0.919 |  |  |  |  |
| Female | 2.4 | (2.2, 2.6) | 2.7 | (2.6, 2.8) | | 0.3 (0.1, 0.5) | | 0.009 | | |  |  |  |  |  |
| Male | 2.6 | (2.4, 2.8) | 2.8 | (2.7, 3.0) | | 0.3 (0.0, 0.5) | | 0.023 | | |  |  |  |  |  |
| Age group (years) (Mean score) | | | | | | | | | | | 0.310 |  |  |  |  |
| 20-29 | 2.3 | (1.9, 2.7) | 2.9 | (2.7, 3.1) | | 0.6 (0.1, 1.0) | | 0.011 | | |  |  |  |  |  |
| 30-39 | 2.5 | (2.2, 2.7) | 2.9 | (2.7, 3.0) | | 0.4 (0.1, 0.7) | | 0.006 | | |  |  |  |  |  |
| 40-49 | 2.6 | (2.4, 2.8) | 2.7 | (2.5, 2.9) | | 0.1 (-0.2, 0.4) | | 0.403 | | |  |  |  |  |  |
| 50-59 | 2.7 | (2.4, 2.9) | 2.8 | (2.6, 3.0) | | 0.1 (-0.2, 0.5) | | 0.499 | | |  |  |  |  |  |
| 60-69 | 2.5 | (2.2, 2.8) | 2.7 | (2.4, 2.9) | | 0.2 (-0.2, 0.5) | | 0.423 | | |  |  |  |  |  |
| Having normal or low blood pressure | | | | | | | | | | | |  |  |  |  |
| Overall |  |  |  |  | |  | |  | | |  |  |  |  |  |
| Disagree (%) | 10.2 | (7.3, 14.0) | 13.0 | (10.8, 15.6) | | 27.5 | |  | | |  |  |  |  |  |
| Neutral (%) | 16.4 | (12.8, 20.7) | 22.4 | (19.6, 25.5) | | 36.6 | | 0.017 | | |  |  |  |  |  |
| Agree (%) | 73.5 | (68.4, 78.0) | 64.6 | (61.2, 67.9) | | –12.1 | |  | | |  |  |  |  |  |
| Mean score | 4.0 | (3.9, 4.2) | 3.8 | (3.7, 3.9) | | -0.2(-0.4, -0.1) | | 0.002 | | |  |  |  |  |  |
| Sex (Mean score) | | | | | | | | | | | 0.647 |  |  |  |  |
| Female | 4.2 | (4.1, 4.4) | 4.0 | (3.9, 4.1) | | -0.3(-0.4, -0.1) | | 0.004 | | |  |  |  |  |  |
| Male | 3.8 | (3.6, 4.0) | 3.6 | (3.5, 3.7) | | -0.2(-0.4, 0.0) | | 0.099 | | |  |  |  |  |  |
| Age group (years) (Mean score) | | | | | | | | | | | 0.485 |  |  |  |  |
| 20-29 | 4.1 | (3.7, 4.4) | 3.9 | (3.8, 4.1) | | -0.1(-0.5, 0.2) | | 0.497 | | |  |  |  |  |  |
| 30-39 | 4.1 | (3.9, 4.3) | 4.0 | (3.9, 4.1) | | -0.1(-0.3, 0.2) | | 0.677 | | |  |  |  |  |  |
| 40-49 | 4.1 | (3.9, 4.3) | 3.8 | (3.6, 4.0) | | -0.3(-0.6, 0.0) | | 0.049 | | |  |  |  |  |  |
| 50-59 | 3.8 | (3.6, 4.1) | 3.5 | (3.3, 3.8) | | -0.3(-0.6, 0.0) | | 0.080 | | |  |  |  |  |  |
| 60-69 | 4.1 | (3.8, 4.4) | 3.7 | (3.4, 3.9) | | -0.4(-0.8, 0.0) | | 0.032 | | |  |  |  |  |  |
| Taking blood pressure medication | | | | | | | | | | | |  |  |  |  |
| Overall |  |  |  |  | |  | |  | | |  |  |  |  |  |
| Disagree (%) | 77.2 | (70.6, 82.8) | 60.4 | (55.9, 64.7) | | –21.8 | |  | | |  |  |  |  |  |
| Neutral (%) | 15.8 | (11.2, 22.0) | 22.0 | (18.5, 25.9) | | 39.2 | | 0.001 | | |  |  |  |  |  |
| Agree (%) | 6.9 | (4.2, 11.3) | 17.6 | (14.4, 21.3) | | 155.1 | |  | | |  |  |  |  |  |
| Mean score | 1.6 | (1.5, 1.8) | 2.2 | (2.1, 2.3) | | 0.6 (0.4, 0.7) | | <0.001 | | |  |  |  |  |  |
| Sex (Mean score) | | | | | | | | | | | 0.009 |  |  |  |  |
| Female | 1.7 | (1.5, 1.8) | 2.0 | (1.8, 2.1) | | 0.3 (0.1, 0.5) | | 0.014 | | |  |  |  |  |  |
| Male | 1.6 | (1.4, 1.8) | 2.4 | (2.2, 2.5) | | 0.8 (0.5, 1.1) | | <0.001 | | |  | 0.79 | (0.51, 1.07) | <0.001 |  |
| Age group (years) (Mean score) | | | | | | | | | | | 0.354 |  |  |  |  |
| 20-29 | 1.6 | (1.3, 2.0) | 2.5 | (2.2, 2.7) | | 0.9 (0.4, 1.3) | | <0.001 | | |  |  |  |  |  |
| 30-39 | 1.9 | (1.5, 2.2) | 2.4 | (2.2, 2.7) | | 0.6 (0.1, 1.1) | | 0.014 | | |  |  |  |  |  |
| 40-49 | 1.4 | (1.2, 1.6) | 2.0 | (1.7, 2.2) | | 0.6 (0.3, 0.9) | | <0.001 | | |  |  |  |  |  |
| 50-59 | 1.7 | (1.4, 2.0) | 2.1 | (1.9, 2.4) | | 0.4 (0.0, 0.8) | | 0.058 | | |  |  |  |  |  |
| 60-69 | 1.6 | (1.3, 1.9) | 1.9 | (1.7, 2.1) | | 0.3 (0.0, 0.7) | | 0.087 | | |  |  |  |  |  |
| Not recommended by healthcare provider | | | | | | | | | | | |  |  |  |  |
| Overall |  |  |  |  | |  | |  | | |  |  |  |  |  |
| Disagree (%) | 27.8 | (22.4, 33.8) | 13.8 | (11.4, 16.8) | | –50.4 | |  | | |  |  |  |  |  |
| Neutral (%) | 17.9 | (12.9, 24.4) | 21.7 | (18.7, 25.0) | | 21.2 | | <0.001 | | |  |  |  |  |  |
| Agree (%) | 54.3 | (47.7, 60.8) | 64.5 | (60.7, 68.1) | | 18.8 | |  | | |  |  |  |  |  |
| Mean score | 3.5 | (3.3, 3.6) | 3.8 | (3.7, 3.9) | | 0.3 (0.1, 0.5) | | 0.001 | | |  |  |  |  |  |
| Sex (Mean score) | | | | | | | | | | | 0.191 |  |  |  |  |
| Female | 3.4 | (3.2, 3.7) | 3.9 | (3.8, 4.0) | | 0.5 (0.2, 0.8) | | 0.001 | | |  |  |  |  |  |
| Male | 3.5 | (3.2, 3.8) | 3.7 | (3.6, 3.8) | | 0.2 (-0.1, 0.5) | | 0.182 | | |  |  |  |  |  |
| Age group (years) (Mean score) | | | | | | | | | | | 0.186 |  |  |  |  |
| 20-29 | 3.4 | (2.9, 3.9) | 3.9 | (3.7, 4.1) | | 0.5 (0.0, 1.0) | | 0.046 | | |  |  |  |  |  |
| 30-39 | 3.3 | (2.9, 3.7) | 3.9 | (3.7, 4.1) | | 0.6 (0.2, 1.1) | | 0.004 | | |  |  |  |  |  |
| 40-49 | 3.6 | (3.3, 3.9) | 3.7 | (3.4, 3.9) | | 0.1 (-0.3, 0.4) | | 0.766 | | |  |  |  |  |  |
| 50-59 | 3.6 | (3.2, 3.9) | 3.6 | (3.4, 3.9) | | 0.0 (-0.4, 0.5) | | 0.833 | | |  |  |  |  |  |
| 60-69 | 3.6 | (3.2, 4.0) | 3.9 | (3.7, 4.1) | | 0.3 (-0.2, 0.8) | | 0.204 | | |  |  |  |  |  |

CI: confidence interval; MD: mean difference; MS: mean score; RPC: relative percentage change, OR: Odd Ratio. Values are weighted. ORs were estimated using survey-weighted logistic regression; linear regression was used for continuous outcomes. **Reference categories were female and age 20–29 years**. Group-Year interaction p-value indicates whether at least one sex/age category differs from 2011 to 2024. OR/Linear Regression ($\hat{\boldsymbol{\beta}}$) provides the change from 2011 to 2024 only amongst categories that are significant. P < 0.01 indicates statistical significance.

**Supplementary Table 3. Attitudes and concern about sodium intake: Changes and group-year interactions, 2011 and 2024.**

|  | **2011 (n=2,603)** | | | | | **2024 (n=3,267)** | | | | | | | | |  | | | | | |  | | | | | |  | | | | |  | | | | |  |  | | | | | | |
| --- | --- | --- | --- | --- | --- | --- | --- | --- | --- | --- | --- | --- | --- | --- | --- | --- | --- | --- | --- | --- | --- | --- | --- | --- | --- | --- | --- | --- | --- | --- | --- | --- | --- | --- | --- | --- | --- | --- | --- | --- | --- | --- | --- | --- |
|  | **%/MS** | | **95% CI** | | | **%/MS** | | | **95% CI** | | | | **RPC/MD** | | | | | | ***P* value** | | | | | | | **Group-Year Interaction**  ***P* value** | | | | | | **OR/** **Linear regression (**$\hat{\boldsymbol{\beta}}$**)** | | | | | **95% CI** | **Interaction**  ***P* value** | | | | | | |
| *Concern about sodium* | | | | | | | | | | | | | | | | | | | | | | | | | |  | | | |  | | | | | |  | | |  | | | | |  |
| Concerned about personal sodium intake | | | |  | | | | | | | | | | | | | | | | | | | | |  | | | |  | | | | |  | | | | | |  | | |  |  |
| Overall (%) | 66.3 | (63.8, 68.7) | | | | 65.9 | | | (64.2, 67.5) | | | -0.5 | | | | | | | 0.812 | | | | | | |  | | | |  | | | | |  | | | |  | |  |  |  |  |
| Sex (%) |  |  | | | |  | | |  | | |  | | | | | | |  | | | | | | | 0.227 | | | |  | | | | |  | | | |  | |  |  |  |  |
| Female | 63.1 | (60.1, 66.0) | | | | 64.6 | | | (62.2, 66.9) | | | 2.3 | | | | | | | 0.449 | | | | | | |  | | | |  | | | | |  | | | |  | |  |  |  |  |
| Male | 69.6 | (65.4, 73.4) | | | | 67.3 | | | (65.0, 69.6) | | | -3.2 | | | | | | | 0.347 | | | | | | |  | | | |  | | | | |  | | | |  | |  |  |  |  |
| Age group (years) (%) |  |  | | | |  | | |  | | |  | | | | | | |  | | | | | | | <0.001 | | | |  | | | | |  | | | |  | |  |  |  |  |
| 20-29 | 55.6 | (46.9, 64.0) | | | | 67.5 | | | (63.7, 71.1) | | | 21.4 | | | | | | | 0.010 | | | | | | |  | | | |  | | | | |  | | | |  | |  |  |  |  |
| 30-39 | 62.5 | (56.9, 67.8) | | | | 67.2 | | | (63.8, 70.5) | | | 7.6 | | | | | | | 0.141 | | | | | | |  | | | |  | | | | |  | | | |  | |  |  |  |  |
| 40-49 | 67.3 | (63.1, 71.2) | | | | 63.1 | | | (59.2, 66.9) | | | -6.2 | | | | | | | 0.144 | | | | | | |  | | | |  | | | | |  | | | |  | |  |  |  |  |
| 50-59 | 73.6 | (69.9, 76.9) | | | | 64.7 | | | (60.6, 68.5) | | | -12.1 | | | | | | | 0.001 | | | | | | |  | | | | 0.66 | | | | | (0.47, 0.91) | | | | 0.005 | |  |  |  |  |
| 60-69 | 73.8 | (69.5, 77.7) | | | | 67.0 | | | (63.3, 70.5) | | | -9.3 | | | | | | | 0.015 | | | | | | |  | | | |  | | | | |  | | | |  | |  |  |  |  |
| Concerned about child's sodium intake | | | | |  | | |  | | |  | | | | | |  | | |  | | | |  | | | | | | |  | | | | | |  |  |  |  |  |  |  |  |
| Overall (%) | 37.1 | | (33.3, 41.0) | | | 56.6 | | | (53.3, 59.8) | | | | | 52.6 | | | | | <0.001 | | | | | | | |  | | | | |  | | | | |  |  | | | | | | |
| Sex (%) |  | |  | | |  | | |  | | | | |  | | | | |  | | | | | | | | 0.216 | | | | |  | | | | |  |  | | | | | | |
| Female | 37.9 | | (33.5, 42.5) | | | 54.1 | | | (49.5, 58.8) | | | | | 42.9 | | | | | <0.001 | | | | | | | |  | | | | |  | | | | |  |  | | | | | | |
| Male | 36.2 | | (29.9, 43.0) | | | 59.0 | | | (54.4, 63.5) | | | | | 63.3 | | | | | <0.001 | | | | | | | |  | | | | |  | | | | |  |  | | | | | | |
| Age group (years) (%) |  | |  | | |  | | |  | | | | |  | | | | |  | | | | | | | | 0.650 | | | | |  | | | | |  |  | | | | | | |
| 20-29 | 42.9 | | (28.1, 59.2) | | | 71.2 | | | (63.3, 78.1) | | | | | 65.9 | | | | | 0.001 | | | | | | | |  | | | | |  | | | | |  |  | | | | | | |
| 30-39 | 39.5 | | (33.0, 46.4) | | | 59.1 | | | (53.6, 64.4) | | | | | 49.6 | | | | | <0.001 | | | | | | | |  | | | | |  | | | | |  |  | | | | | | |
| 40-49 | 33.0 | | (27.9, 38.5) | | | 47.6 | | | (42.0, 53.1) | | | | | 44.0 | | | | | <0.001 | | | | | | | |  | | | | |  | | | | |  |  | | | | | | |
| 50-59 | 34.7 | | (26.7, 43.7) | | | 57.1 | | | (47.7, 66.0) | | | | | 64.5 | | | | | 0.001 | | | | | | | |  | | | | |  | | | | |  |  | | | | | | |
| 60-69 | 43.9 | | (29.7, 59.1) | | | 64.2 | | | (41.7, 81.8) | | | | | 46.3 | | | | | 0.138 | | | | | | | |  | | | | |  | | | | |  |  | | | | | | |
| *Attitudes related to sodium intake* | | | | | | | | | | | | | | | | | | | |  | | | |  | | | | | | |  | | | | | |  |  |  |  |  |  |  |  |
| Perception about personal sodium intake | | | | |  | | |  | | |  | | | | | |  | | |  | | | |  | | | | | | |  | | | | | |  |  |  |  |  |  |  |  |
| Overall |  | |  | | |  | | |  | | | | |  | | | | |  | | | | | | | |  | | | | |  | | | | |  |  | | | | | | |
| Too much (%) | 33.4 | | (31.0, 35.8) | | | 47.5 | | | (45.7, 49.3) | | | | | 42.2 | | | | |  | | | | | | | |  | | | | |  | | | | |  |  | | | | | | |
| The right amount (%) | 57.0 | | (54.4, 59.5) | | | 47.5 | | | (45.7, 49.3) | | | | | –16.7 | | | | | <0.001 | | | | | | | |  | | | | |  | | | | |  |  | | | | | | |
| Too little (%) | 9.7 | | (8.1, 11.5) | | | 5.0 | | | (4.3, 5.9) | | | | | –48.5 | | | | |  | | | | | | | |  | | | | |  | | | | |  |  | | | | | | |
| Mean score | 2.8 | | (2.7, 2.8) | | | 2.5 | | | (2.5, 2.6) | | | | | -0.2 (-0.3, -0.2) | | | | | <0.001 | | | |  | | | | |  | | | | | | | | |  |  | | | |  |  |  |
| Sex (Mean score) | | | | | | | | | | | | | | | | | | | | | | | 0.252 | | | | |  | | | | | | | | |  |  | | | |  |  |  |
| Female | 2.8 | | (2.7, 2.8) | | | 2.5 | | | (2.5, 2.6) | | | | | -0.2 (-0.3, -0.2) | | | | | <0.001 | | | |  | | | | |  | | | | | | | | |  |  | | | |  |  |  |
| Male | 2.7 | | (2.7, 2.8) | | | 2.5 | | | (2.5, 2.6) | | | | | -0.2 (-0.3, -0.1) | | | | | <0.001 | | | |  | | | | |  | | | | | | | | |  |  | | | |  |  |  |
| Age group (years) (Mean score) | | | | | | | | | | | | | | | | | | | | | | | 0.485 | | | | |  | | | | | | | | |  |  | | | |  |  |  |
| 20-29 | 2.8 | | (2.7, 2.9) | | | 2.5 | | | (2.5, 2.6) | | | | | -0.3 (-0.4, -0.1) | | | | | <0.001 | | | |  | | | | |  | | | | | | | | |  |  | | | |  |  |  |
| 30-39 | 2.7 | | (2.6, 2.8) | | | 2.5 | | | (2.4, 2.5) | | | | | -0.2 (-0.3, -0.1) | | | | | <0.001 | | | |  | | | | |  | | | | | | | | |  |  | | | |  |  |  |
| 40-49 | 2.8 | | (2.7, 2.8) | | | 2.5 | | | (2.5, 2.6) | | | | | -0.3 (-0.3, -0.2) | | | | | <0.001 | | | |  | | | | |  | | | | | | | | |  |  | | | |  |  |  |
| 50-59 | 2.8 | | (2.7, 2.8) | | | 2.5 | | | (2.5, 2.6) | | | | | -0.3 (-0.3, -0.2) | | | | | <0.001 | | | |  | | | | |  | | | | | | | | |  |  | | | |  |  |  |
| 60-69 | 2.8 | | (2.7, 2.8) | | | 2.6 | | | (2.5, 2.6) | | | | | -0.2 (-0.3, -0.1) | | | | | <0.001 | | | |  | | | | |  | | | | | | | | |  |  | | | |  |  |  |
| Comparing personal intake with average Canadian | | | | | | |  | | |  | | | | | |  | |  | | | |  | |  | | | | | | | | |  | | | |  |  |  |  |  |  |  |  |
| Overall |  | |  | | |  | | |  | | | | |  | | | | |  | | | | | | | |  | | | | |  | | | | |  |  | | | | | | |
| Lower (%) | 62.5 | | (59.9, 65.0) | | | 51.8 | | | (50.0, 53.6) | | | | | –17.1 | | | | |  | | | | | | | |  | | | | |  | | | | |  |  | | | | | | |
| About the same amount (%) | 25.1 | | (22.9, 27.4) | | | 36.7 | | | (34.9, 38.4) | | | | | 46.2 | | | | | <0.001 | | | | | | | |  | | | | |  | | | | |  |  | | | | | | |
| Higher (%) | 12.4 | | (10.7, 14.5) | | | 11.6 | | | (10.5, 12.8) | | | | | –6.5 | | | | |  | | | | | | | |  | | | | |  | | | | |  |  | | | | | | |
| Mean score | 2.3 | | (2.3, 2.4) | | | 2.5 | | | (2.5, 2.5) | | | | | 0.2 (0.1, 0.2) | | | | | <0.001 | | | | | | | |  | | | | |  | | | | |  |  | | | | | | |
| Sex (Mean score) | | | | | | | | | | | | | | | | | | | | | | | | | | | 0.471 | | | | |  | | | | |  |  | | | | | | |
| Female | 2.3 | | (2.3, 2.4) | | | 2.5 | | | (2.4, 2.5) | | | | | 0.2 (0.1, 0.2) | | | | | <0.001 | | | | | | | |  | | | | |  | | | | |  |  | | | | | | |
| Male | 2.4 | | (2.3, 2.4) | | | 2.6 | | | (2.5, 2.6) | | | | | 0.2 (0.1, 0.3) | | | | | <0.001 | | | | | | | |  | | | | |  | | | | |  |  | | | | | | |
| Age group (years) (Mean score) | | | | | | | | | | | | | | | | | | | | | | | | | | | 0.290 | | | | |  | | | | |  |  | | | | | | |
| 20-29 | 2.5 | | (2.3, 2.7) | | | 2.7 | | | (2.6, 2.8) | | | | | 0.2 (0.0, 0.4) | | | | | 0.032 | | | | | | | |  | | | | |  | | | | |  |  | | | | | | |
| 30-39 | 2.5 | | (2.3, 2.6) | | | 2.6 | | | (2.5, 2.7) | | | | | 0.1 (0.0, 0.3) | | | | | 0.030 | | | | | | | |  | | | | |  | | | | |  |  | | | | | | |
| 40-49 | 2.3 | | (2.3, 2.4) | | | 2.5 | | | (2.5, 2.6) | | | | | 0.2 (0.1, 0.3) | | | | | <0.001 | | | | | | | |  | | | | |  | | | | |  |  | | | | | | |
| 50-59 | 2.2 | | (2.1, 2.3) | | | 2.4 | | | (2.4, 2.5) | | | | | 0.3 (0.2, 0.4) | | | | | <0.001 | | | | | | | |  | | | | |  | | | | |  |  | | | | | | |
| 60-69 | 2.2 | | (2.1, 2.3) | | | 2.3 | | | (2.2, 2.4) | | | | | 0.1 (0.0, 0.2) | | | | | 0.060 | | | | | | | |  | | | | |  | | | | |  |  | | | | | | |
| Belief that sodium reduction improves health | | | | | | |  | | |  | | | | | |  | |  | | | |  | |  | | | | | | | | |  | | | |  |  |  |  |  |  |  |  |
| Overall |  | |  | | |  | | |  | | | | |  | | | | |  | | | | | | | |  | | | | |  | | | | |  |  | | | | | | |
| Disagree (%) | 28.5 | | (26.3, 30.8) | | | 17.8 | | | (16.6, 19.2) | | | | | –37.5 | | | | |  | | | | | | | |  | | | | |  | | | | |  |  | | | | | | |
| Neutral (%) | 31.3 | | (29.0, 33.7) | | | 33.3 | | | (31.7, 34.9) | | | | | 6.0 | | | | | <0.001 | | | | | | | |  | | | | |  | | | | |  |  | | | | | | |
| Agree (%) | 40.2 | | (37.7, 42.8) | | | 48.9 | | | (47.1, 50.6) | | | | | 21.6 | | | | |  | | | | | | | |  | | | | |  | | | | |  |  | | | | | | |
| Mean score | 3.2 | | (3.1, 3.3) | | | 3.4 | | | (3.4, 3.5) | | | | | 0.2 (0.2, 0.3) | | | | | <0.001 | | | | | | | |  | | | | |  | | | | |  |  | | | | | | |
| Sex (Mean score) | | | | | | | | | | | | | | | | | | | | | | | | | | | 0.725 | | | | |  | | | | |  |  | | | | | | |
| Female | 3.1 | | (3.0, 3.2) | | | 3.4 | | | (3.3, 3.4) | | | | | 0.3 (0.2, 0.4) | | | | | <0.001 | | | | | | | |  | | | | |  | | | | |  |  | | | | | | |
| Male | 3.3 | | (3.2, 3.4) | | | 3.5 | | | (3.5, 3.6) | | | | | 0.2 (0.1, 0.3) | | | | | <0.001 | | | | | | | |  | | | | |  | | | | |  |  | | | | | | |
| Age group (years) (Mean score) | | | | | | | | | | | | | | | | | | | | | | | | | | | 0.848 | | | | |  | | | | |  |  | | | | | | |
| 20-29 | 3.2 | | (3.1, 3.4) | | | 3.4 | | | (3.4, 3.5) | | | | | 0.2 (0.0, 0.4) | | | | | 0.068 | | | | | | | |  | | | | |  | | | | |  |  | | | | | | |
| 30-39 | 3.2 | | (3.1, 3.3) | | | 3.5 | | | (3.4, 3.5) | | | | | 0.3 (0.1, 0.4) | | | | | <0.001 | | | | | | | |  | | | | |  | | | | |  |  | | | | | | |
| 40-49 | 3.1 | | (3.0, 3.2) | | | 3.4 | | | (3.3, 3.5) | | | | | 0.3 (0.1, 0.4) | | | | | <0.001 | | | | | | | |  | | | | |  | | | | |  |  | | | | | | |
| 50-59 | 3.2 | | (3.1, 3.3) | | | 3.5 | | | (3.4, 3.6) | | | | | 0.3 (0.1, 0.4) | | | | | <0.001 | | | | | | | |  | | | | |  | | | | |  |  | | | | | | |
| 60-69 | 3.2 | | (3.1, 3.3) | | | 3.4 | | | (3.3, 3.5) | | | | | 0.2 (0.0, 0.3) | | | | | 0.011 | | | | | | | |  | | | | |  | | | | |  |  | | | | | | |

CI: confidence interval; MD: mean difference; MS: mean score; RPC: relative percentage change, OR: Odd Ratio. Values are weighted. ORs were estimated using survey-weighted logistic regression; linear regression was used for continuous outcomes. **Reference categories were female and age 20–29 years**. Group-Year interaction p-value indicates whether at least one sex/age category differs from 2011 to 2024. OR/Linear Regression ($\hat{\boldsymbol{\beta}}$) provides the change from 2011 to 2024 only amongst categories that are significant. P < 0.01 indicates statistical significance.

**Supplementary Table 4. Sodium-related knowledge and misconceptions: Changes and group-year interactions, 2011 and 2024.**

|  | **2011 (n=2,603)** | | **2024 (n=3,267)** | |  |  |  |  |  |  |
| --- | --- | --- | --- | --- | --- | --- | --- | --- | --- | --- |
|  | **%** | **95% CI** | **%** | **95% CI** | **RPC** | ***P* value** | **Group-Year Interaction**  ***P* value** | **OR** | **95% CI** | **Interaction**  ***P* value** |
| Knowing that sodium affects overall health | | | | | | |  |  |  |  |
| Overall (%) | 69.1 | (66.6 , 71.5) | 63.4 | (61.7, 65.1) | -8.2 | <0.001 |  |  |  |  |
| Sex(%) |  |  |  |  |  |  | 0.455 |  |  |  |
| Female | 70.5 | (67.5, 73.3) | 66.1 | (63.8, 68.4) | -6.2 | 0.022 |  |  |  |  |
| Male | 67.6 | (63.5, 71.4) | 60.6 | (58.1, 63) | -10.4 | 0.004 |  |  |  |  |
| Age group (years) (%) |  |  |  |  |  |  | 0.156 |  |  |  |
| 20-29 | 63.1 | (54.3, 71.2) | 62.5 | (58.7, 66.3) | -0.9 | 0.901 |  |  |  |  |
| 30-39 | 69.2 | (63.6, 74.2) | 62.4 | (58.9, 65.9) | -9.7 | 0.042 |  |  |  |  |
| 40-49 | 69.9 | (65.7, 73.7) | 61.7 | (57.8, 65.5) | -11.6 | 0.005 |  |  |  |  |
| 50-59 | 72.6 | (68.6, 76.2) | 61.6 | (57.5, 65.5) | -15.1 | <0.001 |  |  |  |  |
| 60-69 | 70.4 | (65.7, 74.7) | 68.4 | (64.7, 71.8) | -2.9 | 0.491 |  |  |  |  |
| *Associating sodium with:* | | | | | | |  |  |  |  |
| Hypertension |  |  |  |  |  |  |  |  |  |  |
| Overall (%) | 93.9 | (92.5, 95.1) | 82.0 | (80.6, 83.3) | -12.7 | <0.001 |  |  |  |  |
| Sex(%) |  |  |  |  |  |  | 0.361 |  |  |  |
| Female | 94.8 | (93.0, 96.1) | 85.8 | (84.0, 87.4) | -9.5 | <0.001 |  |  |  |  |
| Male | 93.0 | (90.5, 94.8) | 78.0 | (75.9, 79.9) | -16.1 | <0.001 |  |  |  |  |
| Age group (years) (%) |  |  |  |  |  |  | 0.282 |  |  |  |
| 20-29 | 91.9 | (86.4, 95.3) | 70.7 | (67.0, 74.1) | -23.1 | <0.001 |  |  |  |  |
| 30-39 | 92.8 | (88.9, 95.4) | 77.6 | (74.4, 80.4) | -16.4 | <0.001 |  |  |  |  |
| 40-49 | 93.8 | (91.0, 95.7) | 84.8 | (81.7, 87.4) | -9.6 | <0.001 |  |  |  |  |
| 50-59 | 96.5 | (94.7, 97.7) | 87.4 | (84.4, 89.9) | -9.4 | <0.001 |  |  |  |  |
| 60-69 | 94.4 | (91.2, 96.4) | 88.5 | (85.8, 90.7) | -6.3 | 0.004 |  |  |  |  |
| Heart disease |  |  |  |  |  |  |  |  |  |  |
| Overall (%) | 86.2 | (84.2, 88.0) | 73.7 | (72.1, 75.2) | -14.5 | <0.001 |  |  |  |  |
| Sex(%) |  |  |  |  |  |  | 0.115 |  |  |  |
| Female | 39.1 | (36.1, 42.3) | 31.2 | (29.0, 33.5) | -20.3 | <0.001 |  |  |  |  |
| Male | 34.9 | (31.0, 39.1) | 34.1 | (31.8, 36.5) | -2.4 | 0.726 |  |  |  |  |
| Age group (years) (%) |  |  |  |  |  |  | 0.520 |  |  |  |
| 20-29 | 45.0 | (36.5, 53.7) | 37.3 | (33.6, 41.2) | -17.0 | 0.106 |  |  |  |  |
| 30-39 | 41.2 | (35.6, 47.1) | 34.5 | (31.2, 38.0) | -16.3 | 0.046 |  |  |  |  |
| 40-49 | 33.7 | (29.6, 37.9) | 32.1 | (28.5, 35.9) | -4.8 | 0.573 |  |  |  |  |
| 50-59 | 31.8 | (28.0, 35.8) | 29.5 | (25.9, 33.4) | -7.2 | 0.403 |  |  |  |  |
| 60-69 | 33.9 | (29.4, 38.8) | 30.0 | (26.6, 33.6) | -11.6 | 0.187 |  |  |  |  |
| Stroke |  |  |  |  |  |  |  |  |  |  |
| Overall (%) | 78.4 | (76.0, 80.6) | 59.5 | (57.8, 61.2) | -24.2 | <0.001 |  |  |  |  |
| Sex(%) |  |  |  |  |  |  | 0.792 |  |  |  |
| Female | 43.9 | (40.7, 47.1) | 40.0 | (37.6, 42.4) | -8.9 | 0.052 |  |  |  |  |
| Male | 40.1 | (35.9, 44.4) | 39.6 | (37.2, 42.1) | -1.1 | 0.859 |  |  |  |  |
| Age group (years) (%) |  |  |  |  |  |  | 0.447 |  |  |  |
| 20-29 | 46.6 | (38.1, 55.4) | 39.9 | (36.1, 43.9) | -14.4 | 0.164 |  |  |  |  |
| 30-39 | 48.2 | (42.4, 54.1) | 38.9 | (35.4, 42.4) | -19.4 | 0.007 |  |  |  |  |
| 40-49 | 39.3 | (35.1, 43.8) | 40.1 | (36.3, 44.1) | 1.9 | 0.801 |  |  |  |  |
| 50-59 | 37.0 | (33.0, 41.2) | 38.3 | (34.4, 42.4) | 3.4 | 0.663 |  |  |  |  |
| 60-69 | 38.6 | (33.8, 43.6) | 41.7 | (38.0, 45.5) | 8.1 | 0.322 |  |  |  |  |
| Osteoporosis |  |  |  |  |  |  |  |  |  |  |
| Overall (%) | 13.8 | (12.1, 15.5) | 20.5 | (19.2, 22.0) | 48.6 | <0.001 |  |  |  |  |
| Sex(%) |  |  |  |  |  |  | 0.168 |  |  |  |
| Female | 14.9 | (12.9, 17.1) | 20.1 | (18.2, 22.2) | 35.6 | <0.001 |  |  |  |  |
| Male | 12.6 | (10.2, 15.5) | 20.9 | (19.0, 23.0) | 66.1 | <0.001 |  |  |  |  |
| Age group (years) (%) |  |  |  |  |  |  | 0.105 |  |  |  |
| 20-29 | 12.4 | (7.9, 19.0) | 22.1 | (19.0, 25.5) | 77.6 | 0.010 |  |  |  |  |
| 30-39 | 12.9 | (9.7, 16.9) | 22.1 | (19.3, 25.2) | 71.5 | <0.001 |  |  |  |  |
| 40-49 | 17.4 | (14.3, 21) | 20.4 | (17.3, 23.8) | 17.1 | 0.210 |  |  |  |  |
| 50-59 | 14.3 | (11.7, 17.3) | 19.9 | (16.8, 23.4) | 39.4 | 0.011 |  |  |  |  |
| 60-69 | 9.3 | (7.0, 12.3) | 18.3 | (15.5, 21.5) | 97.2 | <0.001 |  |  |  |  |
| Arthritis |  |  |  |  |  |  |  |  |  |  |
| Overall (%) | 37.1 | (34.6, 39.7) | 32.6 | (31.0, 34.2) | -12.1 | <0.001 |  |  |  |  |
| Sex(%) |  |  |  |  |  |  | 0.022 |  |  |  |
| Female | 79.4 | (76.6, 82.0) | 61.5 | (59.1, 63.8) | -22.6 | <0.001 |  |  |  |  |
| Male | 77.3 | (73.4, 80.8) | 57.5 | (55.0, 59.9) | -25.6 | <0.001 |  |  |  |  |
| Age group (years) (%) |  |  |  |  |  |  | 0.731 |  |  |  |
| 20-29 | 71.6 | (63.1, 78.8) | 48.4 | (44.5, 52.4) | -32.3 | <0.001 |  |  |  |  |
| 30-39 | 77.0 | (71.7, 81.6) | 51.3 | (47.7, 54.9) | -33.3 | <0.001 |  |  |  |  |
| 40-49 | 76.9 | (72.8, 80.6) | 61.0 | (57.0, 64.8) | -20.7 | <0.001 |  |  |  |  |
| 50-59 | 83.6 | (80.1, 86.6) | 66.0 | (62.0, 69.8) | -21.0 | <0.001 |  |  |  |  |
| 60-69 | 84.9 | (80.9, 88.1) | 70.1 | (66.5, 73.5) | -17.4 | <0.001 |  |  |  |  |
| Diabetes |  |  |  |  |  |  |  |  |  |  |
| Overall (%) | 38.7 | (36.1, 41.3) | 38.4 | (36.7, 40.1) | -0.8 | 0.854 |  |  |  |  |
| Sex(%) |  |  |  |  |  |  | 0.587 |  |  |  |
| Female | 87.6 | (85.2, 89.6) | 78.5 | (76.4, 80.4) | -10.4 | <0.001 |  |  |  |  |
| Male | 84.8 | (81.5, 87.6) | 68.6 | (66.2, 70.8) | -19.1 | <0.001 |  |  |  |  |
| Age group (years) (%) |  |  |  |  |  |  | 0.295 |  |  |  |
| 20-29 | 84.4 | (76.9, 89.7) | 65.3 | (61.5, 69.0) | -22.6 | <0.001 |  |  |  |  |
| 30-39 | 87.0 | (82.5, 90.4) | 71.0 | (67.6, 74.2) | -18.3 | <0.001 |  |  |  |  |
| 40-49 | 83.8 | (79.9, 87.0) | 71.9 | (68.2, 75.3) | -14.2 | <0.001 |  |  |  |  |
| 50-59 | 88.4 | (85.3, 91.0) | 78.4 | (74.8, 81.6) | -11.4 | <0.001 |  |  |  |  |
| 60-69 | 88.9 | (85.3, 91.7) | 80.9 | (77.7, 83.7) | -9.0 | 0.001 |  |  |  |  |
| Depression |  |  |  |  |  |  |  |  |  |  |
| Overall (%) | 42.0 | (39.4, 44.7) | 39.8 | (38.1, 41.5) | -5.2 | 0.163 |  |  |  |  |
| Sex(%) |  |  |  |  |  |  | 0.283 |  |  |  |
| Female | 38.9 | (35.8, 42.1) | 37.7 | (35.4, 40.1) | -2.9 | 0.576 |  |  |  |  |
| Male | 38.5 | (34.5, 42.7) | 39.1 | (36.7, 41.5) | 1.5 | 0.809 |  |  |  |  |
| Age group (years) (%) |  |  |  |  |  |  | 0.043 |  |  |  |
| 20-29 | 45.8 | (37.2, 54.6) | 39.5 | (35.7, 43.5) | -13.7 | 0.195 |  |  |  |  |
| 30-39 | 42.7 | (37.1, 48.5) | 40.4 | (36.9, 44.0) | -5.3 | 0.510 |  |  |  |  |
| 40-49 | 37.5 | (33.3, 41.9) | 38.9 | (35.1, 42.9) | 3.8 | 0.628 |  |  |  |  |
| 50-59 | 32.0 | (28.2, 36.0) | 36.7 | (32.8, 40.7) | 14.7 | 0.099 |  |  |  |  |
| 60-69 | 35.3 | (30.6, 40.3) | 36.4 | (32.8, 40.1) | 3.2 | 0.717 |  |  |  |  |
| *Sodium information* | | | | | | |  |  |  |  |
| Knowing the maximum recommended sodium intake level | | | | | | |  |  |  |  |
| Overall (%) | 12.4 | (10.8, 14.2) | 21.1 | (19.7, 22.5) | 70.1 | <0.001 |  |  |  |  |
| Sex(%) |  |  |  |  |  |  | 0.631 |  |  |  |
| Female | 12.4 | (10.5, 14.5) | 20.3 | (18.4, 22.3) | 63.8 | <0.001 |  |  |  |  |
| Male | 12.5 | (9.9, 15.6) | 21.9 | (19.9, 24) | 75.4 | <0.001 |  |  |  |  |
| Age group (years) (%) |  |  |  |  |  |  | 0.139 |  |  |  |
| 20-29 | 11.0 | (6.5, 18.0) | 24.3 | (21.1, 27.9) | 121.4 | 0.001 |  |  |  |  |
| 30-39 | 12.7 | (9.2, 17.3) | 25.2 | (22.2, 28.4) | 98.3 | <0.001 |  |  |  |  |
| 40-49 | 12.3 | (9.7, 15.5) | 20.7 | (17.6, 24.1) | 67.9 | <0.001 |  |  |  |  |
| 50-59 | 12.9 | (10.3, 16.1) | 17.3 | (14.4, 20.6) | 33.6 | 0.045 |  |  |  |  |
| 60-69 | 13.5 | (10.5, 17.2) | 17.8 | (15.1, 21.0) | 32.4 | 0.061 |  |  |  |  |
| Correctly interpreting sodium content from Nutrition Facts table | | | | | | |  |  |  |  |
| Overall (%) | 44.4 | (41.8, 47.0) | 55.6 | (53.9, 57.4) | 25.2 | <0.001 |  |  |  |  |
| Sex(%) |  |  |  |  |  |  | 0.788 |  |  |  |
| Female | 45.0 | (41.9, 48.0) | 56.6 | (54.2, 59.0) | 25.9 | <0.001 |  |  |  |  |
| Male | 43.8 | (39.7, 48.0) | 54.6 | (52.2, 57.1) | 24.6 | <0.001 |  |  |  |  |
| Age group (years) (%) |  |  |  |  |  |  | 0.181 |  |  |  |
| 20-29 | 37.9 | (29.8, 46.6) | 52.2 | (48.2, 56.1) | 37.8 | 0.003 |  |  |  |  |
| 30-39 | 46.7 | (41.1, 52.5) | 58.3 | (54.7, 61.8) | 24.7 | 0.001 |  |  |  |  |
| 40-49 | 44.9 | (40.5, 49.3) | 52.8 | (48.8, 56.8) | 17.8 | 0.008 |  |  |  |  |
| 50-59 | 48.8 | (44.6, 53.0) | 55.5 | (51.3, 59.5) | 13.7 | 0.026 |  |  |  |  |
| 60-69 | 42.5 | (37.7, 47.4) | 58.6 | (54.8, 62.3) | 38.0 | <0.001 |  |  |  |  |
| *Sodium-related Misconceptions* | | | | | | |  |  |  |  |
| Believing kosher, sea and gourmet salts to be lower in sodium than table salt | | | | | | |  |  |  |  |
| Overall (%) |  |  |  |  |  |  |  |  |  |  |
| Disagree | 54.2 | (51.6, 56.7) | 43.5 | (41.7, 45.2) | -19.7 |  |  |  |  |  |
| Neutral | 24.4 | (22.3, 26.7) | 32.5 | (30.9, 34.2) | 33.2 | <0.001 |  |  |  |  |
| Agree | 21.4 | (19.4, 23.6) | 24.0 | (22.6, 25.6) | 12.1 |  |  |  |  |  |
| Correctly identifying that kosher, sea and gourmet salts are not lower in sodium than table salt | | | | | | | |  |  |  |
| Sex (%) |  |  |  |  |  |  | 0.847 |  |  |  |
| Female | 53.8 | (50.7, 56.9) | 43.4 | (41.0, 45.8) | -19.4 | <0.001 |  |  |  |  |
| Male | 54.5 | (50.3, 58.6) | 43.5 | (41.1, 46) | -20.1 | <0.001 |  |  |  |  |
| Age group (years) (%) |  |  |  |  |  |  | 0.004 |  |  |  |
| 20-29 | 50.4 | (41.7, 59.0) | 29.3 | (25.9, 33.1) | -41.8 | <0.001 |  | 0.41 | (0.25, 0.68) | <0.001 |
| 30-39 | 53.7 | (47.9, 59.3) | 37.1 | (33.7, 40.7) | -30.8 | <0.001 |  | 0.51 | (0.36, 0.73) | <0.001 |
| 40-49 | 51.6 | (47.2, 56) | 45.0 | (41.1, 49.0) | -12.7 | 0.030 |  |  |  |  |
| 50-59 | 58.6 | (54.5, 62.6) | 50.0 | (45.9, 54.1) | -14.7 | 0.004 |  |  |  |  |
| 60-69 | 58.1 | (53.3, 62.7) | 54.4 | (50.5, 58.2) | -6.4 | 0.236 |  |  |  |  |
| Believing sodium intake is low by not adding salt to food | | | | | | |  |  |  |  |
| Overall (%) |  |  |  |  |  |  |  |  |  |  |
| Disagree | 38.2 | (35.8, 40.7) | 47.3 | (45.6, 49.0) | 23.8 |  |  |  |  |  |
| Neutral | 21.2 | (19.3, 23.3) | 31.2 | (29.7, 32.9) | 47.2 | <0.001 |  |  |  |  |
| Agree | 40.5 | (38.0, 43.1) | 21.5 | (20.1, 22.9) | -46.9 |  |  |  |  |  |
| Correctly identifying that avoiding added salt alone does not mean following a low-sodium diet | | | | | | | |  |  |  |
| Sex (%) |  |  |  |  |  |  | 0.064 |  |  |  |
| Female | 40.1 | (37.1, 43.2) | 52.2 | (49.7, 54.6) | 30.0 | <0.001 |  |  |  |  |
| Male | 36.3 | (32.4, 40.2) | 42.1 | (39.7, 44.6) | 16.2 | 0.014 |  |  |  |  |
| Age group (years) (%) |  |  |  |  |  |  | 0.188 |  |  |  |
| 20-29 | 41.9 | (33.7, 50.4) | 43.6 | (39.8, 47.6) | 4.2 | 0.708 |  |  |  |  |
| 30-39 | 40.4 | (35.0, 46.1) | 48.7 | (45.1, 52.3) | 20.5 | 0.016 |  |  |  |  |
| 40-49 | 37.0 | (32.9, 41.3) | 49.9 | (45.9, 53.9) | 34.8 | <0.001 |  |  |  |  |
| 50-59 | 37.8 | (33.9, 41.9) | 47.1 | (43.0, 51.2) | 24.7 | 0.002 |  |  |  |  |
| 60-69 | 32.5 | (28.2, 37.1) | 46.5 | (42.7, 50.3) | 43.0 | <0.001 |  |  |  |  |
| Believing concern about sodium is unnecessary with low or normal blood pressure | | | | | | |  |  |  |  |
| Overall (%) |  |  |  |  |  |  |  |  |  |  |
| Disagree | 75.9 | (73.6, 78.0) | 59.7 | (58.0, 61.4) | -21.3 |  |  |  |  |  |
| Neutral | 15.6 | (13.7, 17.6) | 25.7 | (24.2, 27.3) | 64.7 | <0.001 |  |  |  |  |
| Agree | 8.6 | (7.3, 10.1) | 14.5 | (13.4, 15.8) | 68.6 |  |  |  |  |  |
| Correctly identifying that sodium is a concern even for individuals with low or normal blood pressure | | | | | | | |  |  |  |
| Sex (%) |  |  |  |  |  |  | 0.035 |  |  |  |
| Female | 76.7 | (74.0, 79.2) | 64.3 | (61.9, 66.6) | -16.1 | <0.001 |  |  |  |  |
| Male | 75.0 | (71.2, 78.5) | 54.9 | (52.5, 57.4) | -26.8 | <0.001 |  |  |  |  |
| Age group (years) (%) |  |  |  |  |  |  | 0.007 |  |  |  |
| 20-29 | 77.6 | (69.5, 84.1) | 48.9 | (45.0, 52.9) | -36.9 | <0.001 |  | 0.28 | (0.15, 0.50) | <0.001 |
| 30-39 | 75.1 | (69.7, 79.8) | 55.7 | (52.1, 59.2) | -25.9 | <0.001 |  | 0.42 | (0.28, 0.62) | <0.001 |
| 40-49 | 74.7 | (70.6, 78.4) | 58.2 | (54.2, 62.0) | -22.1 | <0.001 |  | 0.47 | (0.33, 0.67) | <0.001 |
| 50-59 | 74.3 | (70.5, 77.8) | 65.6 | (61.6, 69.4) | -11.7 | 0.001 |  | 0.66 | (0.47, 0.92) | 0.008 |
| 60-69 | 79.2 | (75.0, 82.9) | 69.2 | (65.5, 72.6) | -12.7 | <0.001 |  | 0.59 | (0.40, 0.86) | 0.002 |

CI: confidence interval; RPC: relative percentage change, OR: Odd Ratio. Values are weighted. ORs were estimated using survey-weighted logistic regression. Reference categories were female and age 20–29 years. Group-Year interaction p-value indicates whether at least one sex/age category differs from 2011 to 2024. OR/Linear Regression ($\hat{\boldsymbol{\beta}}$) provides the change from 2011 to 2024 only amongst categories that are significant. P < 0.01 indicates statistical significance.

**Supplementary Table 5. Behaviours to reduce dietary sodium: Changes and group-year interactions, 2011 and 2024.**

|  | **2011 (n=2,603)** | | **2024 (n=3,267)** | |  |  |  |  |  |  |
| --- | --- | --- | --- | --- | --- | --- | --- | --- | --- | --- |
|  | **2011%** | **95% CI** | **2024%** | **95% CI** | **RPC** | ***P* value** | **Group-Year Interaction**  ***P* value** | **OR** | **95% CI** | **Interaction**  ***P* value** |
| Avoided purchasing or consuming a food due to high sodium content | | | | | | |  |  |  |  |
| Overall (%) | 56.0 | (53.4, 58.7) | 41.4 | (39.6, 43.2) | -26.2 | <0.001 |  |  |  |  |
| Sex (%) |  |  |  |  |  |  | 0.032 |  |  |  |
| Female | 60.5 | (57.3, 63.5) | 42.4 | (39.8, 45) | -29.9 | <0.001 |  |  |  |  |
| Male | 51.4 | (47.1, 55.7) | 40.4 | (37.8, 43) | -21.5 | <0.001 |  |  |  |  |
| Age group (years) (%) |  |  |  |  |  |  | 0.813 |  |  |  |
| 20-29 | 49.6 | (40.8, 58.5) | 38.7 | (34.7, 42.9) | -22.0 | 0.027 |  |  |  |  |
| 30-39 | 59.2 | (53.3, 64.9) | 41.6 | (37.8, 45.4) | -29.8 | <0.001 |  |  |  |  |
| 40-49 | 54.0 | (49.4, 58.4) | 37.5 | (33.5, 41.7) | -30.5 | <0.001 |  |  |  |  |
| 50-59 | 59.3 | (55.1, 63.4) | 43.1 | (38.9, 47.5) | -27.3 | <0.001 |  |  |  |  |
| 60-69 | 59.3 | (54.3, 64.0) | 45.3 | (41.4, 49.3) | -23.5 | <0.001 |  |  |  |  |
| *Cooking and Use of discretionary salt* | | | | | | |  |  |  |  |
| Avoiding salt during cooking |  |  |  |  |  |  |  |  |  |  |
| Overall (%) | 62.3 | (59.8, 64.8) | 43.4 | (41.7, 45.2) | -30.3 | <0.001 |  |  |  |  |
| Sex (%) |  |  |  |  |  |  | 0.670 |  |  |  |
| Female | 61.6 | (58.5, 64.6) | 42.0 | (39.6, 44.5) | -31.8 | <0.001 |  |  |  |  |
| Male | 63.1 | (58.9, 67.1) | 44.9 | (42.4, 47.4) | -28.8 | <0.001 |  |  |  |  |
| Age group (years) (%) |  |  |  |  |  |  | 0.583 |  |  |  |
| 20-29 | 58.3 | (49.4, 66.6) | 39.3 | (35.4, 43.3) | -32.6 | <0.001 |  |  |  |  |
| 30-39 | 57.9 | (52.1, 63.5) | 37.3 | (33.8, 40.9) | -35.5 | <0.001 |  |  |  |  |
| 40-49 | 61.7 | (57.3, 65.9) | 43.9 | (39.9, 47.9) | -28.9 | <0.001 |  |  |  |  |
| 50-59 | 67.6 | (63.6, 71.3) | 44.6 | (40.5, 48.7) | -34.1 | <0.001 |  |  |  |  |
| 60-69 | 67.6 | (62.9, 72.0) | 51.5 | (47.6, 55.3) | -23.9 | <0.001 |  |  |  |  |
| Using spices, herbs and/or seasonings | | | | | | |  |  |  |  |
| Overall (%) | 75.9 | (73.6, 78.1) | 60.5 | (58.8, 62.2) | -20.3 | <0.001 |  |  |  |  |
| Sex (%) |  |  |  |  |  |  | 0.037 |  |  |  |
| Female | 79.7 | (77.0, 82.2) | 61.9 | (59.5, 64.3) | -22.3 | <0.001 |  |  |  |  |
| Male | 72.0 | (68.1, 75.5) | 59.0 | (56.5, 61.4) | -18.1 | <0.001 |  |  |  |  |
| Age group (years) (%) |  |  |  |  |  |  | 0.083 |  |  |  |
| 20-29 | 76.7 | (68.5, 83.3) | 55.0 | (51.0, 59.0) | -28.2 | <0.001 |  |  |  |  |
| 30-39 | 74.0 | (68.4, 79.0) | 59.1 | (55.5, 62.7) | -20.2 | <0.001 |  |  |  |  |
| 40-49 | 77.3 | (73.3, 80.9) | 58.9 | (54.8, 62.8) | -23.9 | <0.001 |  |  |  |  |
| 50-59 | 77.3 | (73.6, 80.6) | 63.6 | (59.5, 67.6) | -17.7 | <0.001 |  |  |  |  |
| 60-69 | 73.2 | (68.5, 77.4) | 65.2 | (61.4, 68.8) | -10.9 | 0.008 |  |  |  |  |
| Avoiding salt at the table | | | | | | |  |  |  |  |
| Overall (%) | 69.2 | (66.7, 71.6) | 58.1 | (56.4, 59.9) | -16.0 | <0.001 |  |  |  |  |
| Sex (%) |  |  |  |  |  |  | 0.092 |  |  |  |
| Female | 72.0 | (69.1, 74.7) | 58.6 | (56.1, 61.0) | -18.6 | <0.001 |  |  |  |  |
| Male | 66.2 | (62.1, 70.2) | 57.7 | (55.2, 60.1) | -13.0 | 0.001 |  |  |  |  |
| Age group (years) (%) |  |  |  |  |  |  | 0.800 |  |  |  |
| 20-29 | 67.7 | (58.8, 75.4) | 55.3 | (51.2, 59.3) | -18.3 | 0.013 |  |  |  |  |
| 30-39 | 66.1 | (60.2, 71.4) | 54.9 | (51.2, 58.5) | -16.9 | 0.002 |  |  |  |  |
| 40-49 | 71.6 | (67.5, 75.3) | 57.9 | (53.9, 61.8) | -19.0 | <0.001 |  |  |  |  |
| 50-59 | 69.3 | (65.3, 73.0) | 58.9 | (54.7, 62.9) | -15.0 | <0.001 |  |  |  |  |
| 60-69 | 71.3 | (66.6, 75.5) | 63.1 | (59.4, 66.8) | -11.4 | 0.007 |  |  |  |  |
| Making own soups, sauces, and salad dressings | | | | | | |  |  |  |  |
| Overall (%) | 57.2 | (54.5, 59.8) | 50.0 | (48.3, 51.8) | -12.5 | <0.001 |  |  |  |  |
| Sex (%) |  |  |  |  |  |  | 0.450 |  |  |  |
| Female | 61.6 | (58.5, 64.7) | 53.3 | (50.8, 55.8) | -13.4 | <0.001 |  |  |  |  |
| Male | 52.5 | (48.2, 56.7) | 46.5 | (43.9, 49.0) | -11.4 | 0.019 |  |  |  |  |
| Age group (years) (%) |  |  |  |  |  |  | 0.034 |  |  |  |
| 20-29 | 44.4 | (35.8, 53.3) | 48.3 | (44.2, 52.4) | 8.7 | 0.433 |  |  |  |  |
| 30-39 | 60.7 | (55.0, 66.2) | 48.8 | (45.1, 52.5) | -19.6 | 0.001 |  |  |  |  |
| 40-49 | 56.7 | (52.2, 61.1) | 45.2 | (41.2, 49.3) | -20.3 | <0.001 |  |  |  |  |
| 50-59 | 61.5 | (57.3, 65.5) | 48.9 | (44.8, 53.1) | -20.4 | <0.001 |  |  |  |  |
| 60-69 | 64.0 | (59.1, 68.6) | 58.2 | (54.4, 62.0) | -9.0 | 0.068 |  |  |  |  |
| Tasting food before adding salt | | | | | | |  |  |  |  |
| Overall (%) | 79.3 | (77.0, 81.4) | 70.5 | (68.9, 72.1) | -11.0 | <0.001 |  |  |  |  |
| Sex (%) |  |  |  |  |  | 0.396 |  |  |  |  |
| Female | 81.0 | (78.2, 83.5) | 74.0 | (71.8, 76.2) | -8.6 |  |  |  |  |  |
| Male | 77.5 | (73.7, 80.9) | 66.9 | (64.4, 69.2) | -13.7 |  |  |  |  |  |
| Age group (years) (%) |  |  |  |  |  | 0.002 |  |  |  |  |
| 20-29 | 75.4 | (66.8, 82.3) | 65.2 | (61.3, 69.0) | -13.4 |  |  |  |  |  |
| 30-39 | 79.8 | (74.6, 84.1) | 67.4 | (63.9, 70.8) | -15.4 |  |  | 0.53 | (0.34, 0.82) | 0.001 |
| 40-49 | 82.0 | (78.1, 85.3) | 71.2 | (67.4, 74.7) | -13.2 |  |  | 0.54 | (0.37, 0.81) | <0.001 |
| 50-59 | 80.8 | (77.2, 83.9) | 69.1 | (65.1, 72.9) | -14.4 |  |  | 0.53 | (0.37, 0.77) | <0.001 |
| 60-69 | 76.9 | (72.5, 80.9) | 78.8 | (75.4, 81.8) | 2.4 |  |  |  |  |  |
| Draining and rinsing canned food | | | | | | | | | | |
| Overall (%) | 57.5 | (54.7, 60.2) | 56.3 | (54.5, 58.1) | -2.1 | 0.468 |  |  |  |  |
| Sex (%) |  |  |  |  |  |  | 0.120 |  |  |  |
| Female | 60.5 | (57.1, 63.7) | 61.8 | (59.3, 64.2) | 2.2 | 0.530 |  |  |  |  |
| Male | 54.4 | (50.0, 58.8) | 50.5 | (47.9, 53.1) | -7.2 | 0.132 |  |  |  |  |
| Age group (years) (%) |  |  |  |  |  |  | 0.383 |  |  |  |
| 20-29 | 59.5 | (50.1, 68.4) | 57.4 | (53.2, 61.4) | -3.7 | 0.671 |  |  |  |  |
| 30-39 | 59.9 | (53.8, 65.7) | 60.3 | (56.6, 63.9) | 0.7 | 0.901 |  |  |  |  |
| 40-49 | 57.3 | (52.5, 62.0) | 53.7 | (49.5, 57.8) | -6.3 | 0.260 |  |  |  |  |
| 50-59 | 58.0 | (53.4, 62.4) | 54.6 | (50.3, 58.9) | -5.7 | 0.297 |  |  |  |  |
| 60-69 | 50.4 | (45.1, 55.8) | 55.1 | (51.1, 59.0) | 9.2 | 0.171 |  |  |  |  |
| *Food selection* | | | | | | |  |  |  |  |
| Requesting no added salt in restaurants | | | | | | |  |  |  |  |
| Overall (%) | 5.1 | (4.2, 6.1) | 13.7 | (12.5, 15.0) | 171.1 | <0.001 |  |  |  |  |
| Sex (%) |  |  |  |  |  |  | 0.008 |  |  |  |
| Female | 5.0 | (3.9, 6.2) | 9.9 | (8.4, 11.5) | 99.4 | <0.001 |  | 2.10 | (1.50, 2.94) | <0.001 |
| Male | 5.2 | (3.8, 7.1) | 17.7 | (15.9, 19.7) | 242.5 | <0.001 |  | 3.95 | (2.63, 5.94) | <0.001 |
| Age group (years) (%) |  |  |  |  |  |  | <0.001 |  |  |  |
| 20-29 | 1.3 | (0.3, 5.8) | 25.0 | (21.7, 28.7) | 1767.9 | <0.001 |  | 24.62 | (3.40, 178.08) | <0.001 |
| 30-39 | 3.5 | (2.0, 6.0) | 15.4 | (12.9, 18.2) | 345.5 | <0.001 |  | 5.08 | (2.25, 11.44) | <0.001 |
| 40-49 | 6.9 | (4.9, 9.7) | 12.8 | (10.3, 15.8) | 85.8 | 0.002 |  |  |  |  |
| 50-59 | 7.7 | (5.7, 10.2) | 9.1 | (6.9, 11.9) | 18.4 | 0.407 |  |  |  |  |
| 60-69 | 5.3 | (3.7, 7.8) | 7.3 | (5.5, 9.8) | 37.5 | 0.187 |  |  |  |  |
| Eating fewer packaged, ready-to-eat foods | | | | | | |  |  |  |  |
| Overall (%) | 69.3 | (66.6, 71.8) | 52.3 | (50.6, 54.1) | -24.5 | <0.001 |  |  |  |  |
| Sex (%) |  |  |  |  |  |  | 0.071 |  |  |  |
| Female | 73.9 | (70.8, 76.7) | 54.5 | (52.1, 57.0) | -26.1 | <0.001 |  |  |  |  |
| Male | 64.6 | (60.3, 68.6) | 50.0 | (47.4, 52.5) | -22.7 | <0.001 |  |  |  |  |
| Age group (years) (%) |  |  |  |  |  |  | 0.053 |  |  |  |
| 20-29 | 58.4 | (49.2, 67.0) | 50.0 | (46.0, 54.0) | -14.4 | 0.097 |  |  |  |  |
| 30-39 | 69.7 | (64.1, 74.8) | 48.6 | (45.0, 52.3) | -30.3 | <0.001 |  |  |  |  |
| 40-49 | 70.3 | (65.9, 74.3) | 48.4 | (44.4, 52.4) | -31.2 | <0.001 |  |  |  |  |
| 50-59 | 74.8 | (70.9, 78.3) | 53.9 | (49.7, 58.0) | -27.9 | <0.001 |  |  |  |  |
| 60-69 | 73.4 | (68.7, 77.7) | 60.3 | (56.4, 64.0) | -17.9 | <0.001 |  |  |  |  |
| Limiting restaurant foods | | | | | | |  |  |  |  |
| Overall (%) | 47.2 | (44.5, 49.8) | 36.7 | (35.0, 38.4) | -22.2 | <0.001 |  |  |  |  |
| Sex (%) |  |  |  |  |  |  | 0.004 |  |  |  |
| Female | 51.1 | (47.9, 54.3) | 36.1 | (33.7, 38.5) | -29.5 | <0.001 |  | 0.54 | (0.45, 0.65) | <0.001 |
| Male | 43.1 | (38.9, 47.3) | 37.4 | (35.0, 39.8) | -13.3 | 0.020 |  |  |  |  |
| Age group (years) (%) |  |  |  |  |  |  | 0.088 |  |  |  |
| 20-29 | 46.1 | (37.4, 55.1) | 40.0 | (36.1, 44.1) | -13.1 | 0.218 |  |  |  |  |
| 30-39 | 45.1 | (39.3, 51.0) | 34.6 | (31.2, 38.1) | -23.4 | 0.002 |  |  |  |  |
| 40-49 | 44.6 | (40.2, 49.1) | 36.8 | (32.9, 40.8) | -17.6 | 0.010 |  |  |  |  |
| 50-59 | 51.5 | (47.2, 55.7) | 33.1 | (29.3, 37.2) | -35.7 | <0.001 |  |  |  |  |
| 60-69 | 49.8 | (44.8, 54.7) | 39.3 | (35.5, 43.2) | -21.0 | 0.001 |  |  |  |  |
| Eating more fresh fruits and vegetables | | | | | | | | | | |
| Overall (%) | 80.6 | (78.4, 82.7) | 67.9 | (66.3, 69.6) | -15.8 | <0.001 |  |  |  |  |
| Sex (%) |  |  |  |  |  |  | 0.410 |  |  |  |
| Female | 84.1 | (81.6, 86.3) | 71.3 | (69.0, 73.5) | -15.2 | <0.001 |  |  |  |  |
| Male | 77.1 | (73.2, 80.6) | 64.4 | (61.9, 66.7) | -16.5 | <0.001 |  |  |  |  |
| Age group (years) (%) |  |  |  |  |  |  | 0.048 |  |  |  |
| 20-29 | 71.4 | (62.6, 78.8) | 66.0 | (62.0, 69.6) | -7.6 | 0.252 |  |  |  |  |
| 30-39 | 82.8 | (78.2, 86.7) | 69.3 | (65.9, 72.6) | -16.3 | <0.001 |  |  |  |  |
| 40-49 | 82.4 | (78.8, 85.5) | 63.5 | (59.5, 67.2) | -23.0 | <0.001 |  |  |  |  |
| 50-59 | 83.2 | (79.8, 86.2) | 67.2 | (63.2, 70.9) | -19.3 | <0.001 |  |  |  |  |
| 60-69 | 83.1 | (79.0, 86.5) | 73.0 | (69.5, 76.3) | -12.2 | <0.001 |  |  |  |  |
| Requesting sauces/dressings on side | | | | | | | | | | |
| Overall (%) | 35.2 | (32.8, 37.6) | 33.6 | (31.9, 35.4) | -4.4 | 0.298 |  |  |  |  |
| Sex (%) |  |  |  |  |  |  | <0.001 |  |  |  |
| Female | 41.4 | (38.4, 44.5) | 33.1 | (30.7, 35.5) | -20.2 | <0.001 |  | 0.70 | (0.58, 0.84) | <0.001 |
| Male | 28.7 | (25.2, 32.4) | 34.2 | (31.8, 36.7) | 19.3 | 0.014 |  |  |  |  |
| Age group (years) (%) |  |  |  |  |  |  | <0.001 |  |  |  |
| 20-29 | 24.4 | (17.8, 32.4) | 36.9 | (33.0, 40.9) | 51.3 | 0.006 |  |  |  |  |
| 30-39 | 26.7 | (22.1, 31.7) | 29.1 | (25.8, 32.5) | 9.0 | 0.4253 |  |  |  |  |
| 40-49 | 38.9 | (34.7, 43.3) | 31.0 | (27.3, 34.9) | -20.3 | 0.007 |  |  |  |  |
| 50-59 | 44.0 | (39.9, 48.2) | 32.3 | (28.4, 36.5) | -26.5 | <0.001 |  | 0.61 | (0.44, 0.85) | 0.001 |
| 60-69 | 42.9 | (38.2, 47.7) | 39.6 | (35.7, 43.6) | -7.8 | 0.293 |  |  |  |  |
|  |  | *Food labels* |  |  |  |  |  |  |  |  |
| Reading Nutrition Facts table to determine sodium content | | | | | | |  |  |  |  |
| Overall (%) | 54.2 | (51.6, 56.8) | 49.6 | (47.9, 51.4) | -8.4 | 0.005 |  |  |  |  |
| Sex (%) |  |  |  |  |  |  | 0.009 |  |  |  |
| Female | 57.5 | (54.4, 60.6) | 48.9 | (46.4, 51.4) | -15.0 | <0.001 |  | 0.71 | (0.59, 0.85) | <0.001 |
| Male | 50.7 | (46.5, 54.9) | 50.4 | (47.9, 52.9) | -0.6 | 0.905 |  |  |  |  |
| Age group (years) (%) |  |  |  |  |  |  | 0.416 |  |  |  |
| 20-29 | 45.1 | (36.4, 54.1) | 47.6 | (43.6, 51.7) | 5.6 | 0.615 |  |  |  |  |
| 30-39 | 54.3 | (48.6, 60.0) | 48.3 | (44.6, 51.9) | -11.2 | 0.081 |  |  |  |  |
| 40-49 | 52.2 | (47.8, 56.6) | 45.5 | (41.6, 49.6) | -12.8 | 0.029 |  |  |  |  |
| 50-59 | 58.7 | (54.5, 62.8) | 49.9 | (45.7, 54.0) | -15.1 | 0.003 |  |  |  |  |
| 60-69 | 63.2 | (58.4, 67.8) | 56.3 | (52.5, 60.1) | -10.9 | 0.026 |  |  |  |  |
| Buying low/reduced sodium products | | | | | | |  |  |  |  |
| Overall (%) | 45.7 | (43.2, 48.4) | 44.6 | (42.9, 46.4) | -2.5 | 0.475 |  |  |  |  |
| Sex (%) |  |  |  |  |  |  | 0.127 |  |  |  |
| Female | 49.9 | (46.7, 53.0) | 46.3 | (43.8, 48.8) | -7.2 | 0.080 |  |  |  |  |
| Male | 41.4 | (37.3, 45.7) | 42.8 | (40.3, 45.3) | 3.4 | 0.578 |  |  |  |  |
| Age group (years) (%) |  |  |  |  |  |  | 0.315 |  |  |  |
| 20-29 | 43.8 | (35.1, 52.9) | 43.0 | (39.1, 47.1) | -1.7 | 0.881 |  |  |  |  |
| 30-39 | 38.9 | (33.4, 44.5) | 44.5 | (40.9, 48.2) | 14.6 | 0.098 |  |  |  |  |
| 40-49 | 43.8 | (39.5, 48.2) | 41.7 | (37.8, 45.8) | -4.7 | 0.490 |  |  |  |  |
| 50-59 | 49.1 | (44.9, 53.3) | 42.7 | (38.7, 46.9) | -13.0 | 0.035 |  |  |  |  |
| 60-69 | 57.5 | (52.6, 62.3) | 50.3 | (46.4, 54.1) | -12.6 | 0.023 |  |  |  |  |
| Looking or would look for healthy choice symbol or logo | | | | | | |  |  |  |  |
| Overall (%) | 29.5 | (27.3, 31.8) | 46.2 | (44.4, 47.9) | 56.4 | <0.001 |  |  |  |  |
| Sex (%) |  |  |  |  |  |  | 0.016 |  |  |  |
| Female | 33.4 | (30.7, 36.3) | 46.9 | (44.5, 49.4) | 40.3 | <0.001 |  |  |  |  |
| Male | 25.5 | (22.1, 29.1) | 45.4 | (42.9, 47.8) | 78.2 | <0.001 |  |  |  |  |
| Age group (years) (%) |  |  |  |  |  |  | 0.116 |  |  |  |
| 20-29 | 23.1 | (16.7, 31.1) | 46.0 | (42.1, 49.9) | 99.2 | <0.001 |  |  |  |  |
| 30-39 | 25.7 | (21.1, 30.9) | 45.0 | (41.4, 48.6) | 74.9 | <0.001 |  |  |  |  |
| 40-49 | 30.7 | (26.8, 34.8) | 43.3 | (39.4, 47.2) | 41.0 | <0.001 |  |  |  |  |
| 50-59 | 32.2 | (28.6, 36.2) | 46.4 | (42.3, 50.5) | 43.9 | <0.001 |  |  |  |  |
| 60-69 | 37.9 | (33.4, 42.6) | 50.1 | (46.3, 53.9) | 32.2 | <0.001 |  |  |  |  |
| Looking or would look for low sodium message or claim | | | | | | |  |  |  |  |
| Overall (%) | 38.8 | (36.4, 41.3) | 49.3 | (47.6, 51.1) | 27.1 | <0.001 |  |  |  |  |
| Sex (%) |  |  |  |  |  |  | 0.102 |  |  |  |
| Female | 42.2 | (39.2, 45.2) | 50.2 | (47.8, 52.7) | 19.1 | <0.001 |  |  |  |  |
| Male | 35.4 | (31.5, 39.4) | 48.3 | (45.9, 50.8) | 36.7 | <0.001 |  |  |  |  |
| Age group (years) (%) |  |  |  |  |  |  | 0.137 |  |  |  |
| 20-29 | 29.8 | (22.2, 38.6) | 45.2 | (41.3, 49.1) | 51.7 | 0.002 |  |  |  |  |
| 30-39 | 37.2 | (31.9, 42.8) | 51.2 | (47.6, 54.8) | 37.7 | <0.001 |  |  |  |  |
| 40-49 | 40.6 | (36.3, 44.9) | 44.7 | (40.8, 48.7) | 10.3 | 0.160 |  |  |  |  |
| 50-59 | 40.4 | (36.4, 44.5) | 48.4 | (44.3, 52.5) | 19.8 | 0.007 |  |  |  |  |
| 60-69 | 48.5 | (43.6, 53.3) | 56.0 | (52.1, 59.7) | 15.5 | 0.017 |  |  |  |  |
| Checking or would check sodium %DV on label | | | | | | |  |  |  |  |
| Overall (%) | 53.6 | (51.0, 56.2) | 50.9 | (49.2, 52.7) | -5.0 | 0.094 |  |  |  |  |
| Sex (%) |  |  |  |  |  |  | 0.408 |  |  |  |
| Female | 54.1 | (51.0, 57.2) | 50.2 | (47.7, 52.6) | -7.3 | 0.051 |  |  |  |  |
| Male | 53.1 | (48.9, 57.2) | 51.8 | (49.3, 54.2) | -2.5 | 0.595 |  |  |  |  |
| Age group (years) (%) |  |  |  |  |  |  | 0.495 |  |  |  |
| 20-29 | 49.1 | (40.4, 57.9) | 49.2 | (45.2, 53.1) | 0.1 | 0.997 |  |  |  |  |
| 30-39 | 58.4 | (52.7, 63.9) | 51.7 | (48.1, 55.3) | -11.5 | 0.051 |  |  |  |  |
| 40-49 | 50.6 | (46.2, 55.0) | 49.3 | (45.3, 53.3) | -2.6 | 0.664 |  |  |  |  |
| 50-59 | 55.8 | (51.7, 59.9) | 49.9 | (45.8, 54.0) | -10.6 | 0.047 |  |  |  |  |
| 60-69 | 54.7 | (49.9, 59.5) | 54.1 | (50.3, 57.9) | -1.1 | 0.841 |  |  |  |  |
| Checking or would check sodium (mg) on label | | | | | | |  |  |  |  |
| Overall (%) | 55.7 | (53.1, 58.2) | 53.3 | (51.6, 55.1) | -4.2 | 0.145 |  |  |  |  |
| Sex (%) |  |  |  |  |  |  | 0.127 |  |  |  |
| Female | 57.7 | (54.6, 60.8) | 53.0 | (50.6, 55.5) | -8.1 | 0.021 |  |  |  |  |
| Male | 53.5 | (49.4, 57.6) | 53.7 | (51.2, 56.1) | 0.3 | 0.950 |  |  |  |  |
| Age group (years) (%) |  |  |  |  |  |  | 0.315 |  |  |  |
| 20-29 | 46.8 | (38.1, 55.7) | 49.7 | (45.7, 53.6) | 6.1 | 0.563 |  |  |  |  |
| 30-39 | 52.7 | (47.0, 58.4) | 52.2 | (48.6, 55.8) | -0.9 | 0.892 |  |  |  |  |
| 40-49 | 55.6 | (51.2, 60.0) | 51.1 | (47.1, 55.1) | -8.2 | 0.134 |  |  |  |  |
| 50-59 | 59.8 | (55.6, 63.8) | 52.5 | (48.4, 56.6) | -12.2 | 0.014 |  |  |  |  |
| 60-69 | 66.2 | (61.5, 70.6) | 60.5 | (56.7, 64.1) | -8.7 | 0.058 |  |  |  |  |

CI: confidence interval; RPC: relative percentage change, OR: Odd Ratio. Values are weighted. ORs were estimated using survey-weighted logistic regression. **Reference categories were female and age 20–29 years**. Group-Year interaction p-value indicates whether at least one sex/age category differs from 2011 to 2024. OR/Linear Regression ($\hat{\boldsymbol{\beta}}$) provides the change from 2011 to 2024 only amongst categories that are significant. P < 0.01 indicates statistical significance.

**Supplementary Table 6. Sodium reduction barriers: Changes and group-year interactions, 2011 and 2024.**

|  | **2011 (n=2,603)** | **95% CI** | **2024 (n=3,267)** | **95% CI** | **RPC/MD** | ***P* value** | **Group-Year Interaction**  ***P* value** | | | | | | **OR/** **Linear regression (**$\hat{\boldsymbol{\beta}}$**)** | | | **95% CI** | | | **Interaction**  ***P* value** | | |  |
| --- | --- | --- | --- | --- | --- | --- | --- | --- | --- | --- | --- | --- | --- | --- | --- | --- | --- | --- | --- | --- | --- | --- |
| Lower sodium foods do not taste as good as regular products | | | | | | | | |  | |  | | |  | | |  | | |  |  |  |
| Overall | | | | | | | | |  | |  | | |  | | |  | | |  |  |  |
| Disagree (%) | 44.0 | (41.4, 46.6) | 24.1 | (22.6, 25.7) | –45.2 | <0.001 | |  | |  | |  | | |  | | |  | | |  |  |
| Neutral (%) | 27.1 | (24.8, 29.6) | 34.2 | (32.6, 35.9) | 26.2 |  |  |  | |  | |  | | |  | | |  | | |  |  |
| Agree (%) | 28.9 | (26.5, 31.3) | 41.6 | (39.9, 43.4) | 44.0 |  |  |  | |  | |  | | |  | | |  | | |  |  |
| Mean score | 2.7 | (2.7, 2.8) | 3.2 | (3.2, 3.3) | 0.5 (0.4, 0.6) | <0.001 | |  | |  | |  | | |  | | |  | | |  |  |
| Sex (Mean score) | | | | | | | |  | | 0.177 | |  | | |  | | |  | | |  |  |
| Female | 2.7 | (2.6, 2.8) | 3.1 | (3.1, 3.2) | 0.4 (0.3, 0.5) | <0.001 | |  | |  | |  | | |  | | |  | | |  |  |
| Male | 2.8 | (2.7, 2.9) | 3.3 | (3.3, 3.4) | 0.5 (0.4, 0.7) | <0.001 | |  | |  | |  | | |  | | |  | | |  |  |
| Age group (years) (Mean score) | | | | | | | |  | | 0.430 | |  | | |  | | |  | | |  |  |
| 20-29 | 2.8 | (2.5, 3.0) | 3.4 | (3.3, 3.4) | 0.6 (0.4, 0.8) | <0.001 | |  | |  | |  | | |  | | |  | | |  |  |
| 30-39 | 2.6 | (2.5, 2.8) | 3.2 | (3.1, 3.2) | 0.5 (0.4, 0.7) | <0.001 | |  | |  | |  | | |  | | |  | | |  |  |
| 40-49 | 2.8 | (2.7, 2.9) | 3.3 | (3.2, 3.4) | 0.5 (0.4, 0.7) | <0.001 | |  | |  | |  | | |  | | |  | | |  |  |
| 50-59 | 2.7 | (2.6, 2.8) | 3.2 | (3.1, 3.3) | 0.4 (0.3, 0.6) | <0.001 | |  | |  | |  | | |  | | |  | | |  |  |
| 60-69 | 2.8 | (2.6, 2.9) | 3.2 | (3.1, 3.2) | 0.4 (0.2, 0.5) | <0.001 | |  | |  | |  | | |  | | |  | | |  |  |
| Price difference between low-sodium and regular foods is too high | | | | | | | |  | |  | |  | | |  | | |  | | |  |  |
| Overall |  |  |  |  |  |  | |  | |  | |  | | |  | | |  | | |  |  |
| Disagree (%) | 65.0 | (62.3, 67.5) | 31.2 | (29.5, 32.9) | –52.0 |  | |  | |  | |  | | |  | | |  | | |  |  |
| Neutral (%) | 21.6 | (19.4, 24.0) | 39.6 | (37.8, 41.4) | 83.3 | <0.001 | |  | |  | |  | | |  | | |  | | |  |  |
| Agree (%) | 13.4 | (11.7, 15.4) | 29.2 | (27.6, 30.9) | 117.9 |  | |  | |  | |  | | |  | | |  | | |  |  |
| Mean score | 2.1 | (2.1, 2.2) | 2.9 | (2.9, 3.0) | 0.8 (0.7, 0.9) | <0.001 | |  | |  | |  | | |  | | |  | | |  |  |
| Sex (Mean score) | | | | | | | |  | | 0.024 | |  | | |  | | |  | | |  |  |
| Female | 2.1 | (2.0, 2.2) | 2.8 | (2.8, 2.9) | 0.7 (0.6, 0.8) | <0.001 | |  | |  | |  | | |  | | |  | | |  |  |
| Male | 2.1 | (2.0, 2.2) | 3.0 | (3.0, 3.1) | 0.9 (0.8, 1.0) | <0.001 | |  | |  | |  | | |  | | |  | | |  |  |
| Age group (years) (Mean score) | | | | | | | |  | | 0.050 | |  | | |  | | |  | | |  |  |
| 20-29 | 2.2 | (2.0, 2.5) | 3.2 | (3.1, 3.2) | 0.9 (0.7, 1.1) | <0.001 | |  | |  | |  | | |  | | |  | | |  |  |
| 30-39 | 2.1 | (1.9, 2.2) | 3.0 | (3.0, 3.1) | 1.0 (0.8, 1.1) | <0.001 | |  | |  | |  | | |  | | |  | | |  |  |
| 40-49 | 2.2 | (2.1, 2.3) | 2.9 | (2.9, 3.0) | 0.8 (0.6, 0.9) | <0.001 | |  | |  | |  | | |  | | |  | | |  |  |
| 50-59 | 2.1 | (2.0, 2.2) | 2.9 | (2.8, 3.0) | 0.8 (0.6, 0.9) | <0.001 | |  | |  | |  | | |  | | |  | | |  |  |
| 60-69 | 2.0 | (1.9, 2.1) | 2.7 | (2.6, 2.8) | 0.7 (0.5, 0.8) | <0.001 | |  | |  | |  | | |  | | |  | | |  |  |
| Lack of time to prepare lower sodium meals from scratch | | | | | | | |  | |  | |  | | |  | | |  | | |  |  |
| Overall |  |  |  |  |  |  | |  | |  | |  | | |  | | |  | | |  |  |
| Disagree (%) | 57.5 | (54.8, 60.1) | 31.5 | (29.9, 33.2) | –45.2 |  | |  | |  | |  | | |  | | |  | | |  |  |
| Neutral (%) | 15.9 | (14.0, 18.0) | 28.5 | (27.0, 30.2) | 79.2 | <0.001 | |  | |  | |  | | |  | | |  | | |  |  |
| Agree (%) | 26.6 | (24.2, 29.1) | 40.0 | (38.2, 41.7) | 50.4 |  | |  | |  | |  | | |  | | |  | | |  |  |
| Mean score | 2.5 | (2.4, 2.5) | 3.1 | (3.0, 3.1) | 0.6 (0.5, 0.7) | <0.001 | |  | |  | |  | | |  | | |  | | |  |  |
| Sex (Mean score) | | | | | | | |  | | 0.688 | |  | | |  | | |  | | |  |  |
| Female | 2.4 | (2.3, 2.5) | 3.0 | (2.9, 3.1) | 0.6 (0.5, 0.7) | <0.001 | |  | |  | |  | | |  | | |  | | |  |  |
| Male | 2.5 | (2.4, 2.6) | 3.1 | (3.1, 3.2) | 0.6 (0.5, 0.8) | <0.001 | |  | |  | |  | | |  | | |  | | |  |  |
| Age group (years) (Mean score) | | | | | | | |  | | 0.002 | |  | | |  | | |  | | |  |  |
| 20-29 | 2.6 | (2.4, 2.9) | 3.5 | (3.4, 3.5) | 0.8 (0.6, 1.1) | <0.001 | |  | |  | | 0.82 | | | (0.56, 1.08) | | | <0.001 | | |  |  |
| 30-39 | 2.5 | (2.4, 2.7) | 3.3 | (3.2, 3.4) | 0.8 (0.6, 1.0) | <0.001 | |  | |  | | 0.78 | | | (0.60, 0.97) | | | <0.001 | | |  |  |
| 40-49 | 2.5 | (2.4, 2.7) | 3.2 | (3.1, 3.3) | 0.6 (0.5, 0.8) | <0.001 | |  | |  | | 0.64 | | | (0.48, 0.80) | | | <0.001 | | |  |  |
| 50-59 | 2.3 | (2.2, 2.4) | 2.9 | (2.8, 3.0) | 0.6 (0.5, 0.8) | <0.001 | |  | |  | | 0.63 | | | (0.48, 0.78) | | | <0.001 | | |  |  |
| 60-69 | 2.2 | (2.1, 2.3) | 2.5 | (2.4, 2.6) | 0.3 (0.2, 0.5) | <0.001 | |  | |  | | 0.33 | | | (0.16, 0.50) | | | 0.001 | | |  |  |
| Lack of support from family/relatives/friends | | | | | | | |  | |  | |  | | |  | | |  | | |  |  |
| Overall |  |  |  |  |  |  | |  | |  | |  | | |  | | |  | | |  |  |
| Disagree (%) | 76.5 | (74.0, 78.9) | 54.0 | (52.1, 55.9) | –29.4 |  | |  | |  | |  | | |  | | |  | | |  |  |
| Neutral (%) | 14.7 | (12.8, 16.8) | 24.7 | (23.2, 26.4) | 68.0 | <0.001 | |  | |  | |  | | |  | | |  | | |  |  |
| Agree (%) | 8.8 | (7.2, 10.7) | 21.2 | (19.8, 22.8) | 140.9 |  | |  | |  | |  | | |  | | |  | | |  |  |
| Mean score | 1.9 | (1.8, 1.9) | 2.4 | (2.3, 2.4) | 0.5 (0.4, 0.6) | <0.001 | |  | |  | |  | | |  | | |  | | |  |  |
| Sex (Mean score) | | | | | | | |  | | 0.032 | |  | | |  | | |  | | |  |  |
| Female | 1.8 | (1.7, 1.9) | 2.2 | (2.2, 2.3) | 0.4 (0.3, 0.5) | <0.001 | |  | |  | |  | | |  | | |  | | |  |  |
| Male | 2.0 | (1.9, 2.1) | 2.5 | (2.5, 2.6) | 0.6 (0.5, 0.7) | <0.001 | |  | |  | |  | | |  | | |  | | |  |  |
| Age group (years) (Mean score) | | | | | | | |  | | <0.001 | |  | | |  | | |  | | |  |  |
| 20-29 | 2.0 | (1.8, 2.2) | 3.0 | (2.9, 3.1) | 0.9 (0.7, 1.2) | <0.001 | |  | |  | | 0.94 | | | (0.69, 1.18) | | | <0.001 | | |  |  |
| 30-39 | 1.9 | (1.8, 2.1) | 2.6 | (2.5, 2.7) | 0.7 (0.5, 0.9) | <0.001 | |  | |  | | 0.70 | | | (0.53, 0.87) | | | <0.001 | | |  |  |
| 40-49 | 1.8 | (1.7, 1.9) | 2.3 | (2.2, 2.4) | 0.5 (0.3, 0.6) | <0.001 | |  | |  | | 0.49 | | | (0.34, 0.63) | | | <0.001 | | |  |  |
| 50-59 | 1.8 | (1.7, 1.9) | 2.1 | (2.0, 2.3) | 0.3 (0.2, 0.5) | <0.001 | |  | |  | | 0.34 | | | (0.20, 0.48) | | | <0.001 | | |  |  |
| 60-69 | 1.8 | (1.6, 1.9) | 1.8 | (1.8, 1.9) | 0.1 (0.0, 0.2) | 0.189 | |  | |  | |  | | |  | | |  | | |  |  |
| Limited or no low sodium options at fast food restaurants | | | | | | | |  | |  | |  | | |  | | |  | | |  |  |
| Overall |  |  |  |  |  |  | |  | |  | |  | | |  | | |  | | |  |  |
| Disagree (%) | 10.4 | (8.7, 12.4) | 7.1 | (6.2, 8.1) | –31.7 |  | |  | |  | |  | | |  | | |  | | |  |  |
| Neutral (%) | 15.7 | (13.7, 17.9) | 20.1 | (18.7, 21.6) | 28.0 | <0.001 | |  | |  | |  | | |  | | |  | | |  |  |
| Agree (%) | 74.0 | (71.3, 76.4) | 72.8 | (71.2, 74.4) | –1.6 |  | |  | |  | |  | | |  | | |  | | |  |  |
| Mean score | 4.0 | (4.0, 4.1) | 4.0 | (4.0, 4.0) | 0.0 (-0.1, 0.0) | 0.478 | |  | |  | |  | | |  | | |  | | |  |  |
| Sex (Mean score) | | | | | | | | | |  | |  | | |  | | |  | | |  |  |
| Female | 4.0 | (4.0, 4.1) | 4.1 | (4.0, 4.1) | 0.0 (-0.1, 0.1) | 0.443 | |  | | 0.078 | |  | | |  | | |  | | |  |  |
| Male | 4.0 | (3.9, 4.1) | 3.9 | (3.9, 4.0) | -0.1 (-0.2, 0.0) | 0.093 | |  | |  | |  | | |  | | |  | | |  |  |
| Age group (years) (Mean score) | | | | | | | |  | | 0.670 | |  | | |  | | |  | | |  |  |
| 20-29 | 4.0 | (3.8, 4.2) | 3.9 | (3.8, 4.0) | -0.1 (-0.3, 0.1) | 0.479 | |  | |  | |  | | |  | | |  | | |  |  |
| 30-39 | 4.0 | (3.8, 4.1) | 4.0 | (3.9, 4.0) | 0.0 (-0.2, 0.2) | 0.953 | |  | |  | |  | | |  | | |  | | |  |  |
| 40-49 | 4.1 | (4.0, 4.2) | 4.0 | (3.9, 4.1) | -0.1 (-0.2, 0.1) | 0.276 | |  | |  | |  | | |  | | |  | | |  |  |
| 50-59 | 4.0 | (3.9, 4.1) | 4.1 | (4.0, 4.2) | 0.1 (-0.1, 0.2) | 0.423 | |  | |  | |  | | |  | | |  | | |  |  |
| 60-69 | 4.2 | (4.0, 4.3) | 4.1 | (4.0, 4.2) | -0.1 (-0.2, 0.1) | 0.423 | |  | |  | |  | | |  | | |  | | |  |  |
| Limited or no low sodium options at sit-down restaurants | | | | | | | |  | |  | |  | | |  | | |  | | |  |  |
| Overall |  |  |  |  |  |  | |  | |  | |  | | |  | | |  | | |  |  |
| Disagree (%) | 13.2 | (11.4, 15.2) | 9.6 | (8.6, 10.8) | –27.3 |  | |  | |  | |  | | |  | | |  | | |  |  |
| Neutral (%) | 21.4 | (19.1, 23.8) | 26.5 | (25.0, 28.2) | 23.8 | <0.001 | |  | |  | |  | | |  | | |  | | |  |  |
| Agree (%) | 65.4 | (62.7, 68.0) | 63.8 | (62.1, 65.6) | –2.4 |  | |  | |  | |  | | |  | | |  | | |  |  |
| Mean score | 3.8 | (3.8, 3.9) | 3.8 | (3.7, 3.8) | 0.0 (-0.1, 0.0) | 0.283 | |  | |  | |  | | |  | | |  | | |  |  |
| Sex (Mean score) | | | | | | | |  | |  | |  | | |  | | |  | | |  |  |
| Female | 3.8 | (3.8, 3.9) | 3.8 | (3.8, 3.9) | 0.0 (-0.1, 0.1) | 0.904 | |  | | 0.204 | |  | | |  | | |  | | |  |  |
| Male | 3.8 | (3.7, 3.9) | 3.7 | (3.7, 3.8) | -0.1 (-0.2, 0.0) | 0.102 | |  | |  | |  | | |  | | |  | | |  |  |
| Age group (years) (Mean score) | | | | | | | |  | | 0.122 | |  | | |  | | |  | | |  |  |
| 20-29 | 3.7 | (3.5, 3.9) | 3.7 | (3.7, 3.8) | 0.1 (-0.1, 0.3) | 0.471 | |  | |  | |  | | |  | | |  | | |  |  |
| 30-39 | 3.8 | (3.7, 3.9) | 3.7 | (3.6, 3.8) | -0.1 (-0.3, 0.0) | 0.166 | |  | |  | |  | | |  | | |  | | |  |  |
| 40-49 | 3.9 | (3.8, 4.0) | 3.7 | (3.6, 3.8) | -0.1 (-0.3, 0.0) | 0.030 | |  | |  | |  | | |  | | |  | | |  |  |
| 50-59 | 3.8 | (3.7, 3.9) | 3.9 | (3.8, 3.9) | 0.1 (-0.1, 0.2) | 0.336 | |  | |  | |  | | |  | | |  | | |  |  |
| 60-69 | 4.0 | (3.8, 4.1) | 3.8 | (3.8, 3.9) | -0.1 (-0.2, 0.0) | 0.120 | |  | |  | |  | | |  | | |  | | |  |  |
| Do not know how to reduce dietary sodium | | | | | | | |  | |  | |  | | |  | | |  | | |  |  |
| Overall |  |  |  |  |  |  | |  | |  | |  | | |  | | |  | | |  |  |
| Disagree (%) | 58.2 | (55.5, 60.8) | 56.1 | (54.4, 57.9) | –3.6 |  | |  | |  | |  | | |  | | |  | | |  |  |
| Neutral (%) | 22.7 | (20.5, 25.0) | 23.4 | (22.0, 24.9) | 3.1 | 0.419 | |  | |  | |  | | |  | | |  | | |  |  |
| Agree (%) | 19.1 | (17.1, 21.4) | 20.5 | (19.1, 21.9) | 7.3 |  | |  | |  | |  | | |  | | |  | | |  |  |
| Mean score | 2.4 | (2.3, 2.4) | 2.4 | (2.4, 2.5) | 0.1 (0.0, 0.1) | 0.156 | |  | |  | |  | | |  | | |  | | |  |  |
| Sex (Mean score) | | | | | | | |  | | 0.200 | |  | | |  | | |  | | |  |  |
| Female | 2.3 | (2.2, 2.4) | 2.3 | (2.2, 2.4) | 0.0 (-0.1, 0.1) | 0.892 | |  | |  | |  | | |  | | |  | | |  |  |
| Male | 2.4 | (2.3, 2.5) | 2.5 | (2.5, 2.6) | 0.1 (0.0, 0.2) | 0.078 | |  | |  | |  | | |  | | |  | | |  |  |
| Age group (years) (Mean score) | | | | | | | |  | | <0.001 | |  | | |  | | |  | | |  |  |
| 20-29 | 2.4 | (2.2, 2.6) | 3.0 | (2.9, 3.1) | 0.6 (0.4, 0.9) | <0.001 | |  | |  | | 0.63 | | | (0.40, 0.87) | | | <0.001 | | |  |  |
| 30-39 | 2.4 | (2.3, 2.5) | 2.6 | (2.5, 2.7) | 0.2 (0.0, 0.3) | 0.048 | |  | |  | |  | | |  | | |  | | |  |  |
| 40-49 | 2.4 | (2.3, 2.5) | 2.4 | (2.3, 2.5) | 0.0 (-0.2, 0.1) | 0.667 | |  | |  | |  | | |  | | |  | | |  |  |
| 50-59 | 2.3 | (2.2, 2.4) | 2.2 | (2.1, 2.3) | -0.1 (-0.2, 0.0) | 0.175 | |  | |  | |  | | |  | | |  | | |  |  |
| 60-69 | 2.3 | (2.2, 2.4) | 2.0 | (1.9, 2.1) | -0.3 (-0.4, -0.1) | <0.001 | |  | |  | | -0.28 | | | (-0.42, -0.14) | | | 0.001 | | |  |  |
| Difficult to understand sodium information on food labels | | | | | | | |  | |  | |  | | |  | | |  | | |  |  |
| Overall |  |  |  |  |  |  | |  | |  | |  | | |  | | |  | | |  |  |
| Disagree (%) | 48.6 | (45.9, 51.2) | 47.0 | (45.2, 48.7) | –3.3 |  | |  | |  | |  | | |  | | |  | | |  |  |
| Neutral (%) | 20.7 | (18.7, 22.8) | 27.9 | (26.4, 29.5) | 34.8 | <0.001 | |  | |  | |  | | |  | | |  | | |  |  |
| Agree (%) | 30.7 | (28.4, 33.2) | 25.1 | (23.6, 26.6) | –18.3 |  | |  | |  | |  | | |  | | |  | | |  |  |
| Mean score | 2.7 | (2.6, 2.8) | 2.6 | (2.6, 2.7) | -0.1 (-0.2, 0.0) | 0.024 | |  | |  | |  | | |  | | |  | | |  |  |
| Sex (Mean score) | | | | | | | |  | | 0.817 | |  | | |  | | |  | | |  |  |
| Female | 2.7 | (2.6, 2.8) | 2.6 | (2.5, 2.6) | -0.1 (-0.2, 0.0) | 0.048 | |  | |  | |  | | |  | | |  | | |  |  |
| Male | 2.7 | (2.6, 2.8) | 2.7 | (2.6, 2.7) | -0.1 (-0.2, 0.0) | 0.202 | |  | |  | |  | | |  | | |  | | |  |  |
| Age group (years) (Mean score) | | | | | | | |  | | <0.001 | |  | | |  | | |  | | |  |  |
| 20-29 | 2.5 | (2.3, 2.7) | 3.0 | (2.9, 3.1) | 0.5 (0.3, 0.7) | <0.001 | |  | |  | | 0.51 | | | (0.28, 0.74) | | | <0.001 | | |  |  |
| 30-39 | 2.7 | (2.5, 2.8) | 2.7 | (2.6, 2.8) | 0.0 (-0.1, 0.2) | 0.740 | |  | |  | |  | | |  | | |  | | |  |  |
| 40-49 | 2.8 | (2.6, 2.9) | 2.6 | (2.5, 2.7) | -0.2 (-0.4, -0.1) | 0.007 | |  | |  | |  | | |  | | |  | | |  |  |
| 50-59 | 2.8 | (2.7, 2.9) | 2.5 | (2.4, 2.6) | -0.3 (-0.5, -0.2) | <0.001 | |  | |  | | -0.34 | | | (-0.49, -0.19) | | | <0.001 | | |  |  |
| 60-69 | 2.8 | (2.7, 2.9) | 2.4 | (2.3, 2.5) | -0.4 (-0.6, -0.2) | <0.001 | |  | |  | | -0.39 | | | (-0.55, -0.23) | | | <0.001 | | |  |  |

CI: confidence interval; MD: mean difference; MS: mean score; RPC: relative percentage change, OR: Odd Ratio. Values are weighted. ORs were estimated using survey-weighted logistic regression; linear regression was used for continuous outcomes. **Reference categories were female and age 20–29 years**. Group-Year interaction p-value indicates whether at least one sex/age category differs from 2011 to 2024. OR/Linear Regression ($\hat{\boldsymbol{\beta}}$) provides the change from 2011 to 2024 only amongst categories that are significant. P < 0.01 indicates statistical significance.
